# Effects of a Multi-Disciplinary Lifestyle Intervention on Cardiometabolic Risk Factors in Young Women with Abdominal Obesity: A Randomised Controlled Trial

**DOI:** 10.1371/journal.pone.0130270

**Published:** 2015-06-26

**Authors:** Bianca L. Share, Geraldine A. Naughton, Philippe Obert, Jennifer K. Peat, Elizabeth A. Aumand, Justin G. Kemp

**Affiliations:** 1 School of Exercise Science, Australian Catholic University, Melbourne, Victoria, Australia; 2 Faculty of Health Sciences, Australian Catholic University, Sydney, NSW, Australia; 3 Murdoch Childrens Research Institute, Melbourne, Victoria, Australia; 4 LAPEC, EA4278, University of Avignon, Avignon, France; Hunter College, UNITED STATES

## Abstract

**Background:**

Young women are under-represented in cardiovascular disease research, with obesity and cardiometabolic risk factor interventions generally targeting older adults. Furthermore, appropriate study designs for young women remain uncertain. This study aimed to assess the impact of a 12 week multi-disciplinary lifestyle intervention on cardiometabolic risk factors in premenopausal women with abdominal obesity.

**Methods:**

Women aged 18–30 y with abdominal obesity [waist circumference (WC) ≥ 80 cm] were randomised to a 12 week lifestyle intervention (n = 26) of physical activity, nutrition education and cognitive behavioural therapy, or a wait-list control group (n = 17). Both groups completed anthropometric, biochemical, nutrition and fitness testing, at pre (0 weeks) and post (12 weeks), with intervention participants completed follow-up testing at 24 weeks.

**Results:**

Results from a linear mixed model showed no between-group differences, other than increased physical activity in the intervention group, at post. In the intervention group alone, positive within-group changes were observed in WC, waist-hip-ratio (WHR), waist-height-ratio (WHtR), resting heart rate, blood pressure, predicted VO_2max_, and total energy intake. Most changes were maintained at 24 weeks post-intervention. Similar within-group improvements were observed in control participants in WC, WHR, WHtR, and systolic blood pressure but no changes were detected in physical activity and nutrition.

**Conclusions:**

Cardiometabolic risk factors were decreased as a result of a lifestyle intervention in young women with abdominal obesity. It is difficult to describe observations in the control group without greater understanding of the behaviour of wait-list participants.

**Trial Registration:**

Australian New Zealand Clinical Trials Registry ACTRN12612001017819

## Introduction

Cardiovascular disease (CVD) represents a major health threat to women worldwide [1], consequently placing substantial burden on public health systems. Most risk factors for CVD, including overweight/obesity and physical inactivity, can be modified through lifestyle interventions [2,3]. The rising prevalence of overweight and obesity is a worldwide concern among young women from developed and developing nations [4]. Currently, 51% of non-hispanic white American women aged 20–39 years are either overweight or obese [5], while prevalence among women in the United Kingdom [6], and Australia [7] aged 25–34 years are 47% and 42%, respectively. An average weight gain of 6 to 12 kg between the ages of 20 to 30 years was noted in a large longitudinal study of women’s health [8], and this weight gain was more than for any other age group [8,9]. Concurrently, sedentary behaviour is increasing in young women [10], with 85% of women aged 18–35 years reporting inactive lifestyles and decreased physical activity [9]. Among women, weight gain is not only a risk factor for CVD but increases the risk of the metabolic syndrome [11], type 2 diabetes mellitus, depression, polycystic ovarian syndrome, infertility and adverse pregnancy outcomes [12].

Despite global strategies for preventive health, there is poor understanding of early risk factors (cardiometabolic risk factors) in young women, and lifestyle interventions can improve health outcomes. Moreover, research assessing the effectiveness of weight management interventions specifically targeting young women is relatively recent [13]. Effective age-appropriate interventions for improving cardiometabolic risk are required for young adults born between 1977 to 1994 (“Generation Y”) who share an urgency for feedback and success [14,15]. The limited research that has been conducted in young overweight/obese women suggests they are difficult to recruit for weight management trials, with high attrition and limited success in losing weight compared with older populations [16]. Furthermore, limited evidence exists to inform the implementation of lifestyle intervention programs targeting young women [17].

Poorer retention but greater success has been reported when results of an online team-based weight loss lifestyle intervention were compared in younger and older adults [17]. However, not all studies of weight loss in young adults following lifestyle interventions report statistical significance [18]. To date, the effectiveness and long-term success of multi-disciplinary lifestyle interventions delivered face-to-face that directly target weight loss in young women remain uncertain [19]. Nonetheless exercise interventions appear to require strong familiarisation of the required physical activity along with some formal contact with the participant. Also, exercise alone is less likely to be effective in weight loss than when combined with some nutrition and psychological support [20].

Therefore, the primary aim of this study was to assess the effectiveness of a lifestyle intervention for reducing CVD risk in young women with abdominal obesity, using a randomised controlled trial (RCT) design. Specifically, the RCT involved a 12-week multi-disciplinary program (physical activity, nutrition education, cognitive behavioural therapy) with Caucasian women aged 18 to 30 years, who shared the cardiometabolic risk factor of abdominal obesity [elevated waist circumference (WC) ≥ 80 cm]. A secondary aim was to examine the effectiveness of the intervention through an improved understanding of the sustainability of any changes. It was hypothesised that the lifestyle intervention would be effective (and sustainable) in reducing cardiometabolic risk in young women with abdominal obesity.

## Materials and Methods

The study protocol was approved by the Australian Catholic University Human Research Ethics Committee (V2009-91) on December 18^th^ 2009 (S1 and S2 Texts). The authors confirm that all ongoing and related trials for this intervention are registered with the Australian New Zealand Clinical Trials Registry (Identifier: ACTRN12612001017819) and the CONSORT reporting guidelines for clinical trials were followed (S1 Table). Data were collected between August 2010 and February 2012. Written informed consent was obtained from all participants.

### Participants

Sixty-two female Caucasian tertiary students at risk of CVD volunteered for this study. Advertisements for recruitment specifically sought young women with abdominal obesity (WC ≥ 80 cm), who were also leading a sedentary lifestyle. Included were women aged 18 to 30 years; with a waist circumference ≥ 80 cm, and who were physically inactive (< 210 minutes per week of organised physical activity in the past six months). Exclusion criteria were being pregnant or breastfeeding; a history of bariatric surgery; and/or having a diagnosis of liver or kidney disease; heart arrhythmia; insulin dependent diabetes mellitus; polycystic ovarian syndrome; thyroid abnormalities. All participants were non-smokers.

**Fig 1:**
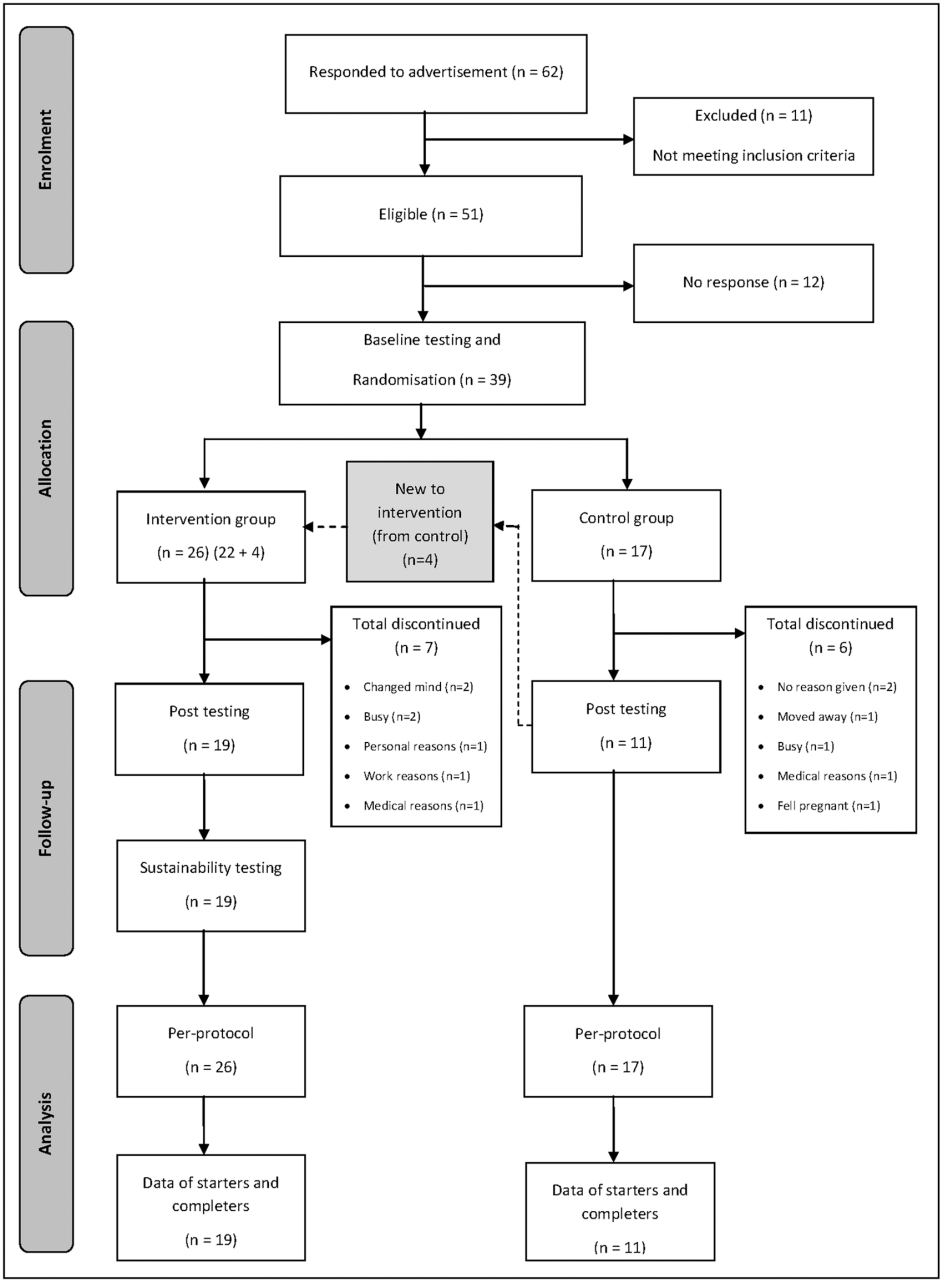
CONSORT participant flow-chart of intervention and control participants.

A power analyses estimated that 18 participants per group would provide the appropriate sample size to detect a large between-subject difference of 1.0 standard deviation (β = 80%, alpha P < 0.05) in waist circumference from pre-intervention to post-intervention. To allow for 20% attrition, there was an attempt to recruit an initial sample size of 44 participants (22 per group). Fig 1 shows the participation of individuals in this study. From 62 women who responded to the recruitment strategy, 11 prospective participants were excluded, and a further 12 did not respond to preliminary contact. Therefore, 39 willing participants underwent pre-intervention/pre-control testing, after which group (block) randomisation occurred via a central administrator who allocated participants to either the intervention group or wait-list (delayed-start) control group. A wait-list control design was chosen because the investigators desired an ethically-sound model which provided all participants with access to the lifestyle intervention. Also, a wait-list control group was considered more appropriate than a passive control group given that health risks were comparable in both groups. After allocation to the wait-list control group for 12 weeks, only four participants continued into the lifestyle intervention phase. Participants were not blinded to their group, but where possible assessors were blinded to group allocation.

### Experimental design

For each testing period (0, 12, 24 weeks), participants attended the laboratory on two occasions. They were requested to refrain from strenuous physical activity in the 24 hours prior to all laboratory sessions. The first visit required the participant to arrive in a fasted state and clinical testing lasted 75 minutes. The second visit of 60 minutes required participants to abstain from caffeine and alcohol for 12 hours.

For the intervention group, testing of cardiometabolic risk factors was performed at pre-intervention (0 weeks), post-intervention (12 weeks) and following a short-term sustainability phase (24 weeks). The control group underwent testing at pre-control and post-control (12 weeks) time periods only. For all testing, measurements were taken at the same time of day (±2 hours) and by the same researcher. For participants in the control group, monthly contact was made to remind them of the control criteria. For the sustainability phase between post-intervention (12 weeks) testing and the following 12 weeks, a sustainability strategy was delivered electronically to the intervention group. This involved a fortnightly newsletter on healthy living tips from evidence-based resources.

Neither the investigators nor the participants were blinded to group allocation as this was considered impractical for the long-term investigation and limited members of the research team. However, to minimise contamination, participants in the intervention group were asked to refrain from disclosing their intervention experience to researchers assigned to data collection and/or analysis and to wait-list control participants. To further minimise bias, assessor blinding occurred within dietary measures and biochemical analyses. For the primary outcome variable of WC, measures were completed in duplicate with reported measures of reliability: coefficient of variation (CV), intraclass correlation coefficient (ICC) and measurement error (ME).

### Testing measures

#### Survey data

A self-administered lifestyle survey was completed to provide data on (i) health status and medical conditions, (ii) nutrition (including alcohol consumption), and (iii) current physical activity habits. The lifestyle survey (S3 Text) was developed specifically for the study and validated using the process of face validation [21].

#### Anthropometric assessment

Body composition was assessed via waist circumference (WC), hip circumference, body mass index (BMI), and body mass. WC was measured to the nearest 0.1 cm in the horizontal plane at the level of the midpoint between the iliac crest and lower costal margin [11]. For WC, CV = 1.26%, ICC (3, 1) = 0.986 and ME = 1.34%, which equates to an error range of ± 2.6 cm. Body mass was measured to the nearest 0.1 kg using digital scales (Tanita, Tokyo, Japan), and height was estimated to the nearest 0.1 cm using a wall-mounted stadiometer (Seca, Germany). BMI was calculated, with overweight/obesity defined as BMI ≥ 25 kg·m^**-2**^ [1]. Waist-to-hip ratio (WHR), and waist-to-height ratio (WHtR) were calculated by dividing participants WC (cm) by their hip circumference (cm) and height (cm), respectively (Browning 2010; WHO, 2000).

*Metabolic syndrome markers and additional biochemical parameters*: Metabolic syndrome was defined according to the most recent and unified criteria [11]. Markers of insulin resistance, including fasting insulin and homeostasis model assessment of insulin resistance (HOMA-IR), and the pro-inflammatory marker high sensitivity C-reactive protein (hs-CRP), were also measured in this study.

Following an overnight fast, intravenous blood was collected Blood lipid profiling of serum concentrations of triglycerides, total cholesterol and high-density lipoprotein (HDL) cholesterol were measured using the Reflotron Plus desktop analyser (Roche, Switzerland). Fasting plasma glucose, insulin and hs-CRP concentrations were analysed by clinical pathology at a leading hospital. Insulin resistance was estimated by HOMA-IR using the equation [22]. For hs-CRP, a value > 3.0 mg·l^-1^ was deemed high risk [23].

After 10 minutes of rest in a quiet, temperature controlled room SBP and DBP were obtained in duplicate from the left arm with an automated digital sphygmomanometer (Carescape V100, Dinamap, GE technology, USA) with the participant in the supine position.

#### Health and fitness evaluations

The YMCA graded submaximal cycle ergometer test [24] was used to estimate aerobic power (predicted VO_2max_), where heart rate was extrapolated against work rate (W) using regression analysis. Physical activity behaviour (S3 Text) was obtained via a 7-day recall [25].

Within the acknowledged limitations of dietary recall [26], 100% of participants completed a three-day food and beverage recall on two consecutive weekdays and on either a Saturday or Sunday of their usual diet. Instructions were provided on how to complete the diary and diagrams of portion sizes were also shown and discussed (S3 Text). Participants were encouraged not to alter their habitual diet during the three day recall period. Macro- and micro-nutrient intakes were analysed by a research dietician, blinded to grouping, using the FoodWorks7 Professional program (Xyris software, Highgate Hill, Queensland, Australia). An estimation of average daily energy intake was also calculated.

### Intervention

The 12-wk lifestyle intervention was comprised of three main components: (1) physical activity (2) nutrition education, and (3) cognitive behavioural therapy (S2 Table). In contrast, participants in the wait-list control group (n = 17) were instructed to continue existing lifestyle choices, and after 12 weeks were invited to complete the lifestyle intervention.

#### Physical activity

Participants undertaking the intervention completed two supervised exercise sessions (progressive circuit training) and one unsupervised, but were prescribed one home-based session (brisk walk or jog) per week. All sessions were devised and administered by a qualified Exercise Scientist who has experience in exercise prescription for elite and healthy populations. The supervised sessions consisted of a general warm-up, a combination of aerobic activities, dynamic strength and/or resistance training, abdominal conditioning, and stretching. Session duration lasted approximately 60 min, with the intensity of the exercise increasing from 6.0 to 8.5 on the OMNI Picture System (ranging from 0 = extremely easy to 10 = extremely hard)[27], by the end of the 12-week period. Intensity was verified during most exercise sessions with a Polar heart rate monitor (Polar Electro, Finland). The home-based, unsupervised training session involved a brisk walk or jog at an RPE of 5–7 on the OMNI Picture System [27]. Participants were encouraged to incorporate intermittent high-intensity intervals into their session. Duration of the session progressed from 30 minutes at intervention commencement to 45 minutes at program completion. As a measure of compliance, participants maintained a detailed training diary including any extra activities they completed during the intervention. The Bruce protocol [28] was completed every three weeks to ensure accuracy of progressive overload of aerobic fitness during the program. Upper (chest-press) and lower (leg-press) body strength was tested via a 5-repetition maximum test to guide the strength and resistance component of the exercise intervention [29]. Sessions occurred in both the gym on campus and at a local park.

#### Nutrition education

Participants in the intervention group received weekly nutrition education sessions guided by a qualified dietician about healthy eating choices from the existing Australian Dietary Guidelines [30]. This information provided education regarding non-dieting weight management and healthy eating principles. Following baseline analysis of a three-day food and beverage recall, nutrition education topics (S2 Table) targeted the perceived needs of the female participants. As such, nutrition was a workshop (educational focus) and did not prescribe a specific caloric intake nor ask participants to monitor their nutritional intake during the intervention.

#### Cognitive behavioural therapy

Within the framework of self-determination theory [31], weekly 60-minute group sessions with a qualified counsellor provided participants with psychosocial support and developed skills to overcome personal barriers to lifestyle change (S2 Table). The program aimed, ultimately, to empower individuals to develop healthier eating and physical activity patterns [32].

### Statistical analyses

Data were analysed using IBM SPSS Statistics, Version 20 for Windows (SPSS Inc, Chicago IL). Data were tested for normal distribution [33], with log transformation performed on data not normally distributed. All data are presented as means ± standard deviation. Statistical significance was set at *P* ≤ 0.05. A linear mixed-model analysis was used to calculate the differences between groups and across time for the intervention and control groups. Hedge’s *g* effect size was used to assess the magnitude of effect. An effect size ≥ 0.2 was considered small, ≥ 0.5 medium, and ≥ 0.8 large [34]. Mean differences and 95% confidence intervals (CI) are reported. An additional linear mixed-model calculation was determined for only the participants in each group who started and completed the study. No differences were found using both models. An independent t-test was used to compare differences between the two groups in changes from baseline to 12 weeks.

## Results

A total of 39 participants were included in the linear mixed-model analysis. For reasons described (Fig 1), 27% of intervention participants and 35% of control participants failed to maintain study involvement beyond pre-intervention/pre-control measures. However, for those completing the intervention, compliance rates were high with 80% attendance at physical activity sessions and 74% at CBT sessions.

### Between group differences

Table 1 shows the results of comparisons between the intervention and control groups before and following the intervention, using a linear mixed model analysis. With only one difference observed between groups at pre-intervention, this supports the homogeneity of the groups. At baseline none of the participants were classified as having the metabolic syndrome [11] however, a baseline difference was found in a higher resting heart rate, in the intervention group, with a moderate effect size (*g* = 0.79). With the exception of WC and weekly physical activity most cardiometabolic risk factors were within normal limits for the population at baseline testing (see footnotes Table 1). At post-intervention, only physical activity was higher in the intervention group than the control group, with a large effect size (*g* = 2.14). Similarly, both the absolute and percentage change in physical activity from pre to post testing were greater for the intervention group, as were the absolute and percentage changes in predicted VO_2max_ when compared to controls.

**Table 1:** Between-group comparisons of cardiometabolic risk factors for the intervention and control group at pre (0 weeks) and post (12 weeks).

	Pre-control/pre-intervention (0 weeks)					Post-control/post-intervention (12 weeks)				
	Control (n = 17)	Intervention (n = 26)	*P* value	Effect size (Hedge’s *g*)	Mean difference (95%CI)	Control (n = 11)	Intervention (n = 19)	*P* value	Effect size (Hedge’s *g*)	Mean difference (95%CI)
Body mass (kg)	86.1 ± 17.8	89.8 ± 21.1	0.564	0.18	3.7 (-9.2 to 16.6)	82.5 ± 19.5	86.9 ± 20.5	0.609	0.21	4.4 (-11.2 to 20.1)
Body mass index (kg·m^-2^)	31.4 ± 6.6	32.2 ± 5.9	0.674	0.13	0.8 (-3.1 to 4.8)	30.0 ± 6.6	31.3 ± 0.9	0.724	0.31	1.3 (-3.5 to 6.0)
Waist circumference (cm)	92.8 ± 10.8	93.1 ± 11.7	0.930	0.03	0.3 (-7.1 to 7.7)	87.2 ± 10.5§	87.3 ± 9.8	0.910	0.01	0.1 (-7.7 to 8.0)
Hip circumference (cm)	113.8 ± 11.5	116.3 ± 13.3	0.537	0.19	2.5 (-5.7 to 10.7)	111.1 ± 11.9	114.2 ± 13.50	0.696	0.23	3.1 (-6.9 to 13.2)
Waist-hip-ratio	0.81 ± 0.03	0.79 ± 0.05	0.242	0.46	0.02 (-0.04 to 0.01)	0.78 ± 0.03§	0.77 ± 0.04	0.581	0.26	-0.01 (-0.05 to 0.01)
Waist-height-ratio	0.56 ± 0.06	0.56 ± 0.06	0.989	0.00	0.0 (-0.04 to 0.04)	0.53 ± 0.06§	0.53 ± 0.05	0.927	0.00	0.0 (-0.04 to 0.04)
Systolic BP (mmHg)	119 ± 8	120 ± 11	0.669	0.10	1.0 (-5.0 to 7.8)	111 ± 12§	116 ± 9	0.312	0.48	5.0 (-2.4 to 13.1)
Diastolic BP (mmHg)	64 ± 8	68 ± 6	0.115	0.10	4.0 (-0.8 to 8.1)	59 ± 5	64 ± 9	0.108	0.62	5.0 (-1.5 to 10.3)
HDL-cholesterol (mM; mg·dL^-1^)	1.7 ± 0.6	1.7 ± 0.5	0.887	0.00	0.0 (-0.4 to 0.3)	2.0 ± 0.5	1.9 ± 0.5	0.706	0.19	-0.1 (-0.4 to 0.3)
	65.6 ± 23.1	65.6 ± 19.3			0.0 (-15.4 to 11.6)	77.2 ± 19.3	73.3 ± 19.3			-3.8 (-15.4 to 11.6)
Triglycerides (mM mg·dL^-1^)#	1.2 ± 0.4	1.3 ± 0.5	0.838	0.21	0.1 (-0.2 to 0.4)	1.5 ± 0.6§	1.4 ± 0.7	0.255	0.15	-0.1 (-0.7 to 0.3)
	106.2 ± 35.4	115.0 ± 44.2			8.8 (-17.7 to 35.4)	132.7 ± 53.1	123.9 ± 61.9			-8.8 (-61.9 to 26.5)
Fasting glucose (mM; mg·dL^-1^)#	4.5 ± 0.6	4.6 ± 0.4	0.353	0.20	0.1 (-0.2 to 0.5)	4.4 ± 0.6	4.6 ± 0.4	0.325	0.40	0.2 (-0.2 to 0.5)
	81.1 ± 10.8	82.9 ± 7.2			1.8 (-3.6 to 9.0)	79.3 ± 10.8	82.9 ± 7.2			3.6 (-3.6 to 9.0)
Total cholesterol (mM mg·dL^-1^)	4.3 ± 0.5	4.4 ± 0.6	0.645	0.17	0.1 (-0.3 to 0.5)	4.3 ± 0.4	4.3 ± 0.8	0.746	0.00	0.0 (-0.5 to 0.6)
	166.0 ± 19.3	169.9 ± 23.1			3.8 (-11.6 to 19.3)	166.0 ± 15.4	166.0 ± 30.9			0.0 (-19,3 to 23.2)
Fasting insulin (mU·l^-1^)#	8.1 ± 4.4	9.4 ± 4.7	0.720	0.28	1.3 (-1.9 to 4.4)	7.4 ± 2.7	8.1 ± 2.6	0.203	0.26	0.7 (-1.3 to 2.8)
HOMA-IR	1.6 ± 1.0	1.9 ± 1.0	0.331	0.29	0.3 (-0.4 to 0.9)	1.4 ± 0.5	1.6 ± 0.5	0.614	0.39	0.2 (-0.2 to 0.6)
hsCRP (mg·l^-1^)#	2.9 ± 2.6	3.5 ± 3.0	0.495	0.21	0.6 (-1.4 to 2.4)	3.9 ± 3.7	4.6 ± 4.9	0.579	0.15	0.6 (-2.9 to 4.2)
Resting heart rate (bpm)	67 ± 10	76 ± 12	0.019*	0.79	9.0 (0.9 to 15.8)	67 ± 9	68 ± 8	0.929	0.12	1.0 (-6.0 to 6.9)
Predicted $\dot{V}O2$max (l·min^-1^)	2.7 ± 0.2	2.4 ± 0.5	0.515	0.74	-0.3 (-0.7 to 0.1)	2.5 ± 0.6	2.8 ± 0.6 £	0.347	0.49	0.3 (-0.2 to 0.7)
Predicted $\dot{V}O2$max (ml·kg^-1^·min^-1^)	32.0 ± 10.3	27.9 ± 7.0	0.133	0.47	-4.1 (-9.8 to 1.5)	31.7 ± 10.9	32.6 ± 6.8 £	0.245	0.10	0.9 (-5.7 to 7.5)
Physical activity (min·week^-1^)#	118 ± 89	97 ± 62	0.870	0.27	21 (-73.4 to 30.9)	121 ± 81	280 ± 67 ¥	< 0.001*	2.14	159 (103.0 to 215.5)
Energy (kj; kcal)	6657 ± 3310	6535 ± 2183	0.945	0.04	122 (-2501 to 2255)	5065 ± 1346	5223 ± 1725	0.743	0.10	158 (-1266 to 1581)
	1591 ± 791	1518 ± 522			29 (-598 to 539)	1210 ± 322	1248 ± 412			38 (-302 to 378)
CHO (g)	194 ± 87	166 ± 74	0.386	0.34	28 (-88.7 to 33.7)	205 ± 101	178 ± 96	0.443	0.27	-27 (-112.1 to 59.0)
Protein (g)#	135 ± 93	173 ± 86	0.097	0.42	38 (-30.1 to 107.6)	137 ± 59	138 ± 63	0.857	0.01	1.0 (-53.9 to 55.0)
Total fat (g)#	66 ± 38	66 ± 34	0.734	0.00	0.0 (-27.9 to 27.6)	50 ± 13	55 ± 14	0.457	0.36	5.0 (-7.0 to 17.0)

Data presented as mean ± standard deviation

^#^ log10 transformation;

*between-group difference, P ≤ 0.05;

^§^ *within-group* difference for control group (pre to post), P ≤ 0.05.

Greater change from baseline to 12 weeks in the intervention versus control group at ¥ P ≤ 0.01 and £ P ≤ 0.05.

BP blood pressure, HDL high-density lipoprotein, HOMA-IR homeostasis model assessment of insulin resistance, hs-CRP high sensitivity C-reactive protein, CHO carbohydrates.

Number of participants who were outside the normal range (see Table 2 for values) for adult women at baseline: BMI n = 35, WC n = 39, WHR n = 16, WHtR n = 25, SBP n = 1, HDL-cholesterol n = 7, triglycerides n = 3, total cholesterol n = 1, HOMA-IR n = 13, hsCRP n = 11, predicted $\dot{V}O2$max (ml·kg^-1^·min^-1^) n = 28, physical activity n = 39.

### Intervention group

For the intervention group, there were positive significant (P ≤ 0.05) changes pre-to post-intervention for WC (-5.8 cm, -6.4%), WHR (-0.02, -2.5%), WHtR (-0.03, -5.5%), SBP (-4 mmHg, -3.4%), DBP (-4.0 mmHg, -5.8%), resting heart rate (-8.0 bpm, -11%), predicted VO_2max_ (+4.7 ml·kg^-1^·min^-1^, +15%), physical activity (+183 min·week^-1^, +97%) and total energy intake (-1312 kj, -22%) (Table 2). Absolute protein intake (g) decreased but this difference disappeared when the decreased total energy intake was accounted for.

**Table 2:** Within-group comparisons of cardiometabolic risk factors for the intervention group at pre-intervention, post-intervention, and sustainability.

				*P*-values			
Variable	Pre-int. (0 weeks) (n = 26)	Post-int. (12 week) (n = 19)	Sustainability (24 week) (n = 19)	Pre-int. vs Post-int.	Pre-int. vs Sustainability	Post-int. vs Sustainability	Population norms/range for adult women
Body mass (kg)	89.8 ± 21.1	86.9 ± 20.5	86.1 ± 20.3	0.791	0.408	0.369	-
Body mass index (kg·m^-2^)	32.2 ± 5.9	31.3 ± 0.9	31.0 ± 6.1	0.447	0.197	0.291	18.5–24.9 [1]
Waist circumference (cm)	93.1 ± 11.7	87.3 ± 9.8	87.8 ± 9.4	< 0.001*	0.002*	0.696	≤ 80 [11]
Hip circumference (cm)	116.3 ± 13.3	114.2 ± 13.50	114.5 ± 13.9	0.309	0.627	0.734	-
Waist-hip-ratio	0.79 ± 0.05	0.77 ± 0.04	0.77 ± 0.04	0.002*	0.018*	0.998	< 0.80 [1]
Waist-height-ratio	0.56 ± 0.06	0.53 ± 0.05	0.53 ± 0.05	< 0.001*	0.001*	0.841	< 0.50 [35]
Systolic BP (mmHg)	120 ± 11	116 ± 9	116 ± 11	0.047*	0.131	0.967	≤ 130 [11]
Diastolic BP (mmHg)	68 ± 6	64 ± 9	64 ± 6	0.040*	0.050*	0.841	≤ 85 [11]
HDL-cholesterol (mM; mg·dL^-1^)	1.7 ± 0.5	1.9 ± 0.5	1.9 ± 0.4	0.193	0.386	0.726	≥ 1.29 [11]
	65.6 ± 19.3	73.5 ± 19.3	73.5 ± 15.4				≥ 49.8
Triglycerides (mM; mg·dL^-1^)#	1.3 ± 0.5	1.4 ± 0.7	1.5 ± 1.0	0.855	0.271	0.221	≤ 1.7 [11]
	115.0 ± 44.2	123.9 ± 61.9	132.7 ± 88.5				≤ 150.4
Fasting glucose (mM; mg·dL^-1^)#	4.6 ± 0.4	4.6 ± 0.4	4.6 ± 0.5	0.728	0.559	0.756	≤ 5.6 [11]
	82.9 ± 7.2	82.9 ± 7.2	82.9 ± 9.0				≤ 100.9
Total cholesterol (mM;mg·dL^-1^)	4.4 ± 0.6	4.3 ± 0.8	4.3 ± 0.6	0.542	0.671	0.937	< 5.5 [36]
	169.9 ± 23.1	166.0 ± 30.9	166.0 ± 23.1				< 212.3
Fasting insulin (mU·l^-1^)#	9.4 ± 4.7	8.1 ± 2.6	8.8 ± 4.7	0.957	0.466	0.344	-
HOMA-IR	1.9 ± 1.0	1.6 ± 0.5	1.80 ± 0.97	0.176	0.575	0.480	< 2.0 [37]
hsCRP (mg·l^-1^)#	3.5 ± 3.0	4.6 ± 4.9	4.7 ± 3.8	0.743	0.148	0.140	< 3.0 [23]
Resting heart rate (bpm)	76 ± 12	68 ± 8	66 ± 9	0.020*	0.004*	0.489	-
Predicted $V˙O_{2}$max (l·min^-1^)	2.4 ± 0.5	2.8 ± 0.6	2.5 ± 0.6	0.029*	0.471	0.179	-
Predicted $V˙O_{2}$max (ml·kg^-1^·min^-1^)	27.9 ± 7.0	32.6 ± 6.8	30.9 ± 9.7	0.000*	0.248	< 0.001*	≥ 31.0 [38]
Physical activity (min·week^-1^)#	97 ± 62	280 ± 67	143.7 ± 48.4	< 0.001*	0.002*	< 0.001*	≥ 210 [39]
Energy (kj; kcal)	6535 ± 2183	5223 ± 1725	5538 ± 2588	0.007*	0.269	0.161	-
	1562 ± 522	1248 ± 412	1324 ± 618				
CHO (g)	166 ± 74	178 ± 96	154 ± 83	0.638	0.928	0.638	-
Protein (g)#	173 ± 86	138 ± 63	150 ± 86	0.012*	0.520	0.079	-
Total fat (g)#	66 ± 34	55 ± 14	56 ± 20	0.227	0.575	0.513	-

Data presented as mean ± standard deviation

^#^ log_10_ transformation

* P ≤ 0.05.

Int, intervention. BP blood pressure, HDL high-density lipoprotein, HOMA-IR homeostasis model assessment of insulin resistance, hs-CRP high sensitivity C-reactive protein, CHO carbohydrates.

Many of the improvements observed at post-intervention were maintained at sustainability (24 weeks) testing including, WC, WHR, WHtR, DBP and resting heart rate (Table 2). The aforementioned cardiometabolic markers were significant from pre-intervention to sustainability testing (i.e. maintenance had occurred) but no further improvements were seen between post-testing to sustainability testing. In fact, predicted VO_2max_ and physical activity reduced during the 12-week sustainability phase however, physical activity remained greater than at pre-intervention.

### Control group

Despite being requested to maintain normal lifestyle habits, the control group displayed several improvements from pre- to post-testing, including WC (-5.6 cm, -6.2%), WHR (-0.03, -3.8%), WHtR (-0.03, -5.5%) and SBP (-8.0 mmHg, -7.0%), while circulating triglycerides rose (Table 2). In contrast to the intervention group, there were no changes in reported DBP, reported physical activity, predicted VO_2max_, resting heart rate and energy intake following the 12-week control period.

## Discussion

The effectiveness of the multi-disciplinary lifestyle intervention for reducing CVD risk in young women was highlighted by within-group improvements in a range of risk factors for the intervention group, with several of these improved markers retained 12 weeks after completion of the lifestyle intervention. Thus, these data suggest a relatively successful intervention for reducing CVD risk with promising sustainability. However, when between-group comparisons were made with the control group, the findings suggested a research design that was largely unsuccessful in identifying the effectiveness of the intervention phase. This raises several concerns associated with a wait-list control design when used with overweight/obese young women. Concerns from the current study support previous findings that describe more difficulties in retaining younger than older females to research trials [13].

Positive changes within the intervention group were demonstrated with improvements in WC related measures, systolic and diastolic blood pressure, aerobic fitness and physical activity, and dietary energy intake. Collectively, these measures imply that the intervention produced cardiovascular, more so than metabolic, benefits for the intervention group. The use of investigative procedures such as non-invasive echocardiography or MRI may provide insight into the significance of these changes [40].

Improvements in fitness and physical activity and reduced energy intake also suggest that the exercise and nutrition education components of the intervention, respectively, were effective. Moreover, there was strong evidence for sustainability of intervention-induced improvements at 24 weeks, suggesting that the CBT component produced positive behavioural change in participants. It has been shown that poor adherence to behavioural programs is a barrier to successful long-term weight maintenance beyond the completion of the intervention [41]. In the present study, adherence to the physical activity component and attendance at the CBT sessions was high amongst intervention participants. This might explain the success in maintenance of some cardiometabolic risk factors observed during the short-term sustainability phase. Additionally, it has been suggested that improvements in maintenance might be achieved through incorporating technology to monitor weight, physical activity and behaviour [42]. This type of innovation could be easily integrated into a population of young adults.

However, similar post-intervention changes in our wait-list control group, made it difficult to detect any anthropometric, biochemical, fitness or dietary differences between groups following the 12-week intervention phase. The control group in this RCT also displayed a decrease in WC that paralleled the intervention group, despite undetectable changes in self-reported physical activity and nutrition. The decreased WC of the control group is particularly difficult to explain without further investigative procedures such as accelerometry for physical activity and more rigorous dietary monitoring, but does suggest that basic awareness of CVD risk might be enough to evoke change in targeted populations [43,44].

Randomised controlled trials provide the highest level of evidence for the effects of an intervention and are deemed to be scientifically rigorous [45], with control groups employed to provide a contrast for the experimental group [46] and for establishing the efficacy of an intervention [47]. But changed outcomes that arise from a wait-list control condition can be detrimental rather than beneficial to a randomised controlled trial [46], and this occurred in the current study. Therefore, wait-list control designs might not be appropriate for this population.

Retention and compliance of wait-list participants is also an issue for consideration when planning control conditions essential for maintaining the rigour of a randomised control design. In this study, more than one-third of the control group failed to return for post-testing at 12 weeks despite researcher attempts to maintain contact. In contrast, once engaged in the intervention group, retention to lifestyle change was high for at least 24 weeks. It is postulated that assigning participants to the control group decreased their motivation to participate. Thus, the duration (12 weeks) and/or conditions (no changes to existing lifestyle) of the control group do not appear suitable for women of this age group and demographic. Alternative strategies for immediate engagement, perhaps via topics of interest using multimedia such as health-related *apps* or support groups might improve commitment and maintain control group compliance. There were also some difficulties encountered with recruitment, with almost a quarter of interested and eligible participants failing to engage after initial commitment (prior to group allocation). Complexities associated with recruiting young women for weight management trials, especially from ‘Generation Y’, may result in smaller sample sizes and require shorter periods of engagement [16].

Although not always the case [48], successful outcomes have been observed following lifestyle interventions with middle-aged [2] and older women [3]. Furthermore, a large scale success of weight loss has been observed in a recent eight year study of adults aged 45–65 years showing an 8.5% mean body weight loss after year one of a lifestyle program [49]. Subsequent monitoring indicated maintenance of approximately 4–5% over the 7 years. However difficulties in the external validity amongst younger adults (21–44 years) was acknowledged [19]. Moreover, there is a lack of effective lifestyle interventions for young adults, with no weight loss programs to date developed specifically to address the needs of this age group. Outcomes, enrolment and retention rates have been compared between younger (18–35 years, n = 21) and more mature (> 35 years, n = 277) adults (66% female) engaged in similar behavioural weight loss and physical activity programs [15]. Results showed attendance was 30% lower in young adults and they were 30% less likely to be retained for the 6-month assessment. Weight loss and increases in total physical activity from baseline to 6 months were significantly less in the younger population. Although the number of younger adults was relatively small, these results indicated that traditional interventions were less successful in young adults [15]. These findings are supported by results from other studies attempting to engage young adults [50–52].

This study is not without limitations. Despite recruitment and retention strategies, the sample size, particularly in the control condition, was lower than anticipated at completion of the study, suggesting it was slightly under-powered. Additionally, the results are specific to Caucasian women at a tertiary institution. To capture any potential changes to control groups in a wait-list design, objective measures (e.g. accelerometers, fortnightly anthropometric measures) might be useful. In addition, the use of more objective measures of physical activity and dietary compliance would strengthen evidence of change in this age group of women. In agreement with previous reports, not all food and activity diaries were completed with precision. Future researchers may benefit from the use of diet quality changes rather than diet intake.

Nonetheless, the study contributes to a very limited number of healthy lifestyle interventions in young adult women (< 30 years of age) with cardiometabolic risk factors [50]. The multi-disciplinary lifestyle intervention confirms the potential value in health changes observed post-intervention, with favourable sustainability at a 12 weeks.

## Conclusions

Within-group analysis showed that the multi-disciplinary lifestyle intervention comprising physical activity, nutrition education and CBT was positive for the reduction of CVD risk factors both immediately after and beyond the completion of the program. However, we also observed positive, but unexpected and difficult to explain, changes in the wait-list control group. Therefore, comparative lifestyle benefits for the intervention group may have been masked by undetectable weight management behaviour in the control group. When considered alongside the difficulties faced with recruitment and retention, especially in the nature of the control group, these results provide a challenge for prospective study designs with young women with cardiometabolic risk factors. Traditional RCT designs may be problematic for healthy lifestyle interventions in young women.

## Supporting Information

S1 Table CONSORT checklist. (DOC)

S2 Table Lifestyle intervention: Outline of the 12-week multi-disciplinary lifestyle intervention of physical activity, nutrition education and cognitive behavioural therapy. (DOC)

S1 Text Application for ethical approval of research projects with human participants. (DOC)

S2 Text Summary of research for ethics application. (DOC)

S3 Text Lifestyle survey. (DOC)
