# Supplementary material for: Effects of a Multi-Disciplinary Lifestyle Intervention on Cardiometabolic Risk Factors in Young Women with Abdominal Obesity: A Randomised Controlled Trial
Source: PLoS One. 2015 Jun 26;10(6):e0130270. doi: 10.1371/journal.pone.0130270 (PMC4483260; doi:10.1371/journal.pone.0130270)
Supplement: S2 Table — (DOC) [file pone.0130270.s002.doc]

| **Week** | **Physical Activity** | | | **Nutrition Education** | **Cognitive Behavioural Therapy (CBT)** |
| --- | --- | --- | --- | --- | --- |
|  | **Supervised session intensity (%)** | **Unsupervised session duration (mins)** | **Fitness testing** | **Weekly Topics** | **Weekly Topics** |
| **1** | 60 | 30  30  30 | Bruce, 5-RM | Australian dietary guidelines for adults | Group formation and introductions |
| **2** | 60 |  |  | Australian physical activity guidelines for adults | Changing Behaviour: Benefits of change |
| **3** | 65 |  |  | Label reading and interpretation | Identifying strengths: Building momentum |
| **4** | 65 | 35  35  35 | Bruce, 5-RM | Serving size | Motivation: Goal setting for success |
| **5** | 70 |  |  | Glycemic index | Overcoming the barriers to change |
| **6** | 70 |  |  | Separating fact from fiction | Food for health: Making nutrition work for you |
| **7** | 75 | 40  40  40 | Bruce, 5-RM | Food variety, dietary fibre and snack choices | Letting go of the ‘uncontrollable’ |
| **8** | 75 |  |  | Dietary fats and take-away food | Examining self-talk and building self-confidence |
| **9** | 80 |  |  | Fluids, hydration and alcohol | Physical activity and mood |
| **10** | 80 | 45  45  45 | Bruce, 5-RM | Protein and iron | Social support |
| **11** | 85 |  |  | Fad diets and body image | Behavioural change for life |
| **12** | 85 |  |  | Dairy products and calcium | Group endings |

**S2 Table: Lifestyle intervention:** Outline of the 12-week multi-disciplinary lifestyle intervention of physical activity, nutrition education and cognitive behavioural therapy.

*5-RM* repetition max
