# Supplementary material for: Effects of a Multi-Disciplinary Lifestyle Intervention on Cardiometabolic Risk Factors in Young Women with Abdominal Obesity: A Randomised Controlled Trial
Source: PLoS One. 2015 Jun 26;10(6):e0130270. doi: 10.1371/journal.pone.0130270 (PMC4483260; doi:10.1371/journal.pone.0130270)
Supplement: S1 Text — (DOC) [file pone.0130270.s003.doc]

| 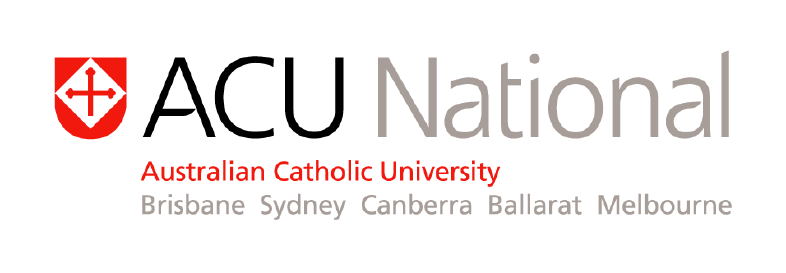Human Research Ethics Committee | ***Office Use Only***  HREC Register No.: |
| --- | --- |
|  |  |
|  | Principal Investigator's or Supervisor's Name:  Dr Justin Kemp |
|  | Students' Names:  Miss Bianca Louise Share |

| **PROJECT TITLE:** | The metabolic syndrome in young women |
| --- | --- |
| **SIMPLE TITLE FOR USE ON PARTICIPANT DOCUMENTS:** | The metabolic syndrome in young women |

**Application for Ethics Approval**

# Research Projects with Human Participants

All research projects involving human participants and/or access to their records/files/specimens must be approved by the University’s Human Research Ethics Committee (HREC).

1. **Before completing this form, applicants should read carefully the document *Ethical Conduct in Research Involving Humans: Guidelines for Applicants to the Human Research Ethics Committee*** (approved by Academic Board 23 February 2001). This document is available on the Research Services website at [www.acu.edu.au/research](http://www.acu.edu.au/research). All applications are assessed in accordance with the National Statement available at:

<http://www.nhmrc.gov.au/publications/synopses/e72syn.htm>

1. Completed applications are to be emailed as an attachment to: res.ethics@acu.edu.au. Once the application has been checked by the relevant Research Services Officer, you will be required to submit a signed hard copy.
2. Applications must be submitted at least six (6) weeks before the proposed date of commencement of the research project.

[Please note that any information of a commercial or patentable nature should be forwarded separately and marked “COMMERCIAL IN CONFIDENCE”.]

In preparing your application please note the following:

- ALL sections and subsections must be answered. Incomplete applications will be returned to the Investigator or Supervisor without being considered by the HREC.
- Insofar as possible, the *Information Letter to Participants* and the *Consent Form* are to be formulated in plain English. **There should be no typographical, spelling or grammatical errors.**

Sample *Information Letter to Participants* and sample *Consent Forms* can be found at www.acu.edu.au/research

If you require assistance in interpreting the Guidelines, or if you have any queries, please contact any member of HREC and/or any Research Services Officer. .

***You are reminded that contact with participants and/or access to their records/files/specimens must not commence until written ethics approval has been received from the HREC.***

| **SECTION A: ISSUES RELATING TO HUMAN PARTICIPANTS** |
| --- |

|  | **Does your research involve any of the following?** (Please tick) | **YES** | **NO** |
| --- | --- | --- | --- |
|  |  |  |  |
| **A.0** | **Use of non-identifiable data about human beings that is use of existing collections of data or records that contain only non-identifiable data about human beings** |  |  |
|  | If your answer to the above question is “Yes” go to Section B1 and complete B1, B2.1, C1 only and attach a copy of the Research proposal.  YOUR PROPOSAL ONLY REQUIRES TO BE RECORDED AND DOES NOT NEED ETHICS CLEARANCE |  |  |
|  |  |  |  |
|  | **IF YOU ANSWER “YES” TO ANY OF THE QUESTIONS A.01 TO A.08 YOUR RESARCH PROJECT MUST BE REVIEWED BY THE FULL COMMITTEE.** |  |  |
| **A.01** | **(Chapter 3.3 of *National Statement*) Interventions and Therapies, including Clinical and non-clinical trials and innovations. Does your research involve** |  |  |
|  | - 1. Administration of any substance or agent |  |  |
|  | 01.2 A treatment or diagnostic procedure |  |  |
|  | 01.3 A surgical procedure |  |  |
|  | - 1. Any other therapeutic procedure or devices, preventative procedure or diagnostic device or procedure |  |  |
| **A.02** | **(Chapter 3.4 of the *National Statement*) Human Genetics. Does your research involve** |  |  |
|  | - 1. Study of single or multiple genes, gene-gene interaction or gene-environment interaction |  |  |
|  | 02.2 Acquired somatic variation or inherited gene sequences |  |  |
|  | 02.3 Gene expressions or genes of individuals, families or populations |  |  |
|  | - 1. Epigenetics or use of informatics and genetic information or clinical phenotypes |  |  |
| **A.03** | **(Chapter 3.6 of the *National Statement*) Human Stem Cell Research. Does your research involve** |  |  |
|  | - 1. Use of embryonic or somatic stem cells or those derived from primordial germ cells |  |  |
| **A.04** | **(Chapter 4.1 of the National Statement) Women who are pregnant and the human foetus. Does your research involve** |  |  |
|  | 04.1 Research on a woman who is pregnant and the foetus *in utero* |  |  |
|  | 04.2 Research on the separated human foetus or on foetal tissue |  |  |
| **A.05** | **(Chapter 4.4 of the National Statement) People highly dependent on medical care who may be unable to give consent. Does your research involve** |  |  |
|  | 05.1 People who are highly dependent on medical care |  |  |
|  | 05.2 People in terminal care, emergency care or intensive care |  |  |
|  | - 1. People who are unconscious or in a state of post-traumatic coma unresponsiveness |  |  |
| **A.06** | **(Chapter 4.5 of the National Statement) People with a cognitive impairment, an intellectual disability or a mental illness. Does your research involve** |  |  |
|  | - 1. Anyone who is intellectually, mentally or physically impaired |  |  |
| **A.07** | **(Chapter 4.6 of the National Statement) People who may be involved in illegal activities. Does your research involve** |  |  |
|  | 07.1 Study that intends to expose illegal activity |  |  |
|  | 07.2 The likelihood of discovering illegal activity, even if not intended |  |  |
|  | 07.3 The inadvertent and unexpected discovery of illegal activity |  |  |
|  |  | **YES** | **NO** |
| **A.08** | **(Chapter 4.7 of the National Statement) Aboriginal and Torres Strait Islander Peoples. your research must conform to the Values and Ethics – Guidelines for Ethical Conduct in Aboriginal and Torres Strait Islander Health Research document. All applications must be referred to the ACU Indigenous unit prior to submission to the Research Office. Does your research involve** |  |  |
|  | 08.1 Aboriginal and Torres Strait Islander Peoples |  |  |
|  |  |  |  |
|  | **The following questions are designed to help you and the Committee ascertain the level of risk involved in the project and factors that may affect consent** |  |  |
|  |  |  |  |
| **A.1** | **Access to members of the following groups who may be vulnerable or unable to give fully informed consent:** |  |  |
|  | - 1. Minors (anyone under the age of 18, e.g., students or children) |  |  |
|  | - 1. Anyone at risk of criminal or civil liability, damage to financial or social standing or to employability |  |  |
|  | - 1. Elderly people who may be vulnerable or unable to give fully informed consent |  |  |
|  | - 1. Welfare recipients who may be vulnerable |  |  |
|  | - 1. Members of minority groups who may be vulnerable or unable to give fully informed consent |  |  |
|  | - 1. Anyone who is a prisoner or ward of the State |  |  |
|  | - 1. Other: (please state) |  |  |
|  |  |  |  |
| **A.2** | **Risk of social, mental or physical harm:** |  |  |
|  | 1. Access to confidential data (including student data, patient or client data) without the participant’s written consent |  |  |
|  | 1. Performance of any acts which might diminish self-esteem or cause embarrassment or distress |  |  |
|  | 1. Use of non-treatment or placebo control conditions |  |  |
|  | 1. Collection of body tissues or fluid samples |  |  |
|  | 1. Administration of any stimuli, tasks, investigations or procedures which may be experienced by participants as physically or mentally stressful, painful, noxious, aversive or unpleasant, either during or following research procedures |  |  |
|  | 1. Any possibility of cardio-pulmonary difficulties (e.g., asthma, headaches, shortness of breath, chest pains, heart attack) |  |  |
|  | 1. Treatments or techniques with unpleasant or harmful side effects |  |  |
|  | 1. Contact with electrical supply (e.g., electrical stimulation) |  |  |
|  | 1. Use of injections which may result in the transmission of HIV (AIDS) or another disease |  |  |
|  |  | **YES** | **NO** |
|  | 1. Intended contact with persons with infectious diseases (e.g., measles, hepatitis, TB, whooping cough) |  |  |
|  | 1. Other: (please state) |  |  |
|  |  |  |  |
| **A.3** | **Possible breaches of State or Commonwealth legislation:** |  |  |
|  | 1. Interviews/Focus Groups involving the photographing or audio/video-taping of participants |  |  |
|  | 1. Deception of participants |  |  |
|  | 1. Possibility of identifying participant/s, either directly or indirectly, through identifiers or by deduction |  |  |
|  | 1. Disclosure of participants' identity to anyone other than the investigators at any stage |  |  |
|  | 1. Use of one or more fertilised ova |  |  |
|  | 1. Finger-printing or DNA "finger-printing” of participants |  |  |
|  | 1. Recombinant DNA, ionizing radiation, or contact with hazardous, illegal or restricted substances (e.g., chemicals, quarantinable materials) |  |  |
|  | 1. Other: (please state) |  |  |
| **A.4** | **Secondary use of existing human specimens:** |  |  |
|  | 1. Access to human pathology or diagnostic specimens (e.g., blood sera or tissue samples) originally provided to authorities for purposes other than those sought in your research project |  |  |
|  |  |  |  |
| **A.5** | **An application for funding either internal or external to the University** |  |  |
|  |  |  |  |

|  |  | |  |  |
| --- | --- | --- | --- | --- |
| **A.6** | **Level of Risk** | |  |  |
|  | Please indicate the level of risk to the participant in this research. | |  |  |
|  |  | “Negligible risk” is defined as follows: “Negligible risk research describes research in which there is no foreseeable risk of harm or discomfort, and any foreseeable risk is no more than inconvenience. Examples of inconvenience may include filling in a form, participating in a street survey, or giving up time to participate in research. (*National Statement*, 2007, p.16) | | |
|  |  | "Low risk" is defined as follows: "low risk research describes research in which the only foreseeable risk is one of discomfort. Discomfort can involve body and/or mind and include, for example, minor side effects of medication, the discomforts related to measuring blood pressure, and anxiety induced by an interview.” (*National Statement,* 2007, p.16). | | |
|  |  | "More than low risk" refers to "Research in which the risk for participants is more serious than discomfort.” This could include research with potential for physical or psychological harms, devaluation of personal worth, social harms, including damage to social networks, economic harms and legal harms.. (*National Statement*, 2007, p.16) | | |
|  | [For information on HREC procedures when considering applications see *Guidelines*, Part A, Section 3.] | |  |  |
| **A.7** |  | **Peer Review of the Research**  The National Statement states that where prior peer review has judged that a project has research merit, the question of its research merit is no longer subject to the judgement of those ethically reviewing the research (*National Statement, 2007, s.*1.2) | **YES** | **NO** |
|  | A.7.1 | Has the project been peer reviewed? |  |  |
|  | A7.2 | If Yes, please indicate what process was used to review the project  1. Extensive consultations have occurred prior to the development of this project with researchers internal and external to ACU.  2. This project has successfully passed a PhD Candidature Confirmation presentation to an assessment panel and other members of the School's staff. |  |  |

| **SECTION B: GENERAL INFORMATION** |
| --- |

|  |  | | | | | | | | |  | | |
| --- | --- | --- | --- | --- | --- | --- | --- | --- | --- | --- | --- | --- |
| **B.1** | **Principal Supervisor** | | | | | | | | | (delete inapplicable title) | | |
|  | **Title & Full Name:**  Dr Justin Kemp | | | | | | | **School:**  Exercise Science | | | | |
|  | **Qualifications:**  PhD, MSc, BEd | | | | | | | **Campus:**  Melbourne (St. Patrick's) | | | | |
|  | **Full Postal Address:**  Australian Catholic University  Faculty of Health Sciences  Locked Bag 4115 Fitzroy MDC VIC 3065 | | | | | | | **Telephone No:**  9953 3031 | | | | **Fax No:**  9953 3095 |
|  |  |  |  |  |  |  |  | **E-mail address:**  justin.kemp@acu.edu.au | | | | |
|  |  | | | | | | |  | | | | |
|  |  | | | | | | |  | | | | |
|  | **Co-Supervisor** | | | | | | | | | (delete inapplicable title) | | |
|  | **Title & Full Name:**  Professor Geraldine Naughton | | | | | | | **School:**  Exercise Science | | | | |
|  | **Qualifications:**  PhD | | | | | | | **Campus:**  Melbourne (St. Patrick's) | | | | |
|  | **Postgraduate or Undergraduate** (if student)**:**  N/A | | | | | | | **Current Enrolment Programme:**  N/A | | | | |
|  | **Full Postal Address:**  Australian Catholic University  Faculty of Health Sciences  Locked Bag 4115 Fitzroy MDC VIC 3065 | | | | | | | **Telephone No:**  9953 3034 | | | | **Fax No:**  9953 3095 |
|  |  |  |  |  |  |  |  | **E-mail address:**  geraldine.naughton@acu.edu.au | | | | |
|  |  | | | | | | |  | | | | |
|  |  | | | | | | |  | | | | |
|  | **Student Researcher** | | | | | | | | | (delete inapplicable title) | | |
|  | **Title & Full Name:**  Miss Bianca Louise Share | | | | | | | **School:**  Exercise Science | | | | |
|  | **Qualifications:**  Bachelor of Exercise Science (Honours) | | | | | | | **Campus:**  Melbourne (St.Patrick's) | | | | |
|  | **Postgraduate or Undergraduate** (if student)**:**  Post-graduate | | | | | | | **Current Enrolment Programme:**  PhD | | | | |
|  | **Full Postal Address:**  Australian Catholic University  Faculty of Health Sciences  Locked Bag 4115 Fitzroy MDC VIC 3065 | | | | | | | **Telephone No:**  9953 3225 | | | | **Fax No:**  9953 3095 |
|  |  |  |  |  |  |  |  | **E-mail address:**  bianca.share@acu.edu.au | | | | |
|  |  | | | | | | |  | | | | |
|  |  | | | | | | |  | | | | |
|  | **Associate Supervisor** | | | | | | | | | (delete inapplicable title) | | |
|  | **Title & Full Name:**  Professor Philippe Obert | | | | | | | **School:**  Faculté des Sciences | | | | |
|  | **Qualifications:**  PhD | | | | | | | **Campus:**  N/A | | | | |
|  | **Postgraduate or Undergraduate** (if student)**:**  N/A | | | | | | | **Current Enrolment Programme:**  N/A | | | | |
|  | **Full Postal Address:**  Universite` d’Avignon  33 Louise Street Avignon France 84000 | | | | | | | **Telephone No:**  +33 490162930 | | | | **Fax No:**  N/A |
|  |  |  |  |  |  |  |  | **E-mail address:**  philippe.obert@univ-avignon.fr | | | | |
|  |  | | | | | | |  | | | | |
|  |  | | | | | | |  | | | | |
|  | **Co-Investigator** | | | | | | | | | (delete inapplicable title) | | |
|  | **Title & Full Name:**  Ms Elizabeth Aumand | | | | | | | **School:**  Exercise Science | | | | |
|  | **Qualifications:**  Bachelor of English/Psychology;  Masters of Ex&SpPsy; Masters of Counselling | | | | | | | **Campus:**  Melbourne (St. Patrick's) | | | | |
|  | **Postgraduate or Undergraduate** (if student)**:**  N/A | | | | | | | **Current Enrolment Programme:**  N/A | | | | |
|  | **Full Postal Address:**  Australian Catholic University  Faculty of Health Sciences  Locked Bag 4115 Fitzroy MDC VIC 3065 | | | | | | | **Telephone No:**  9953 3164 | | | | **Fax No:**  9953 3095 |
|  |  |  |  |  |  |  |  | **E-mail address:**  elizabeth.aumand@acu.edu.au | | | | |
|  |  | | | | | | |  | | | | |
|  |  | | | | | | |  | | | | |
|  | **Co-Investigator 5 (if staff) or Student Researcher 5** | | | | | | | | | (delete inapplicable title) | | |
|  | **Title & Full Name:** | | | | | | | **School:** | | | | |
|  | **Qualifications:** | | | | | | | **Campus:** | | | | |
|  | **Postgraduate or Undergraduate** (if student)**:** | | | | | | | **Current Enrolment Programme:** | | | | |
|  | **Full Postal Address:** | | | | | | | **Telephone No:** | | | | **Fax No:** |
|  |  |  |  |  |  |  |  | **E-mail address:** | | | | |
|  |  | | | | | | |  | | | | |
|  |  | | | | | | |  | | | | |
| **B.2** | **Duration of Project** | | | | | | |  | | | | |
|  | - 1. Anticipated duration of project as a whole | | | | | | |  | | | | |
|  | From: DD/MM/YY | | | | 05.01.09 | | | To: DD/MM/YY | | | 31.12.11 | |
|  | - 1. Anticipated duration of data collection | | | | | | |  | | | | |
|  | From: DD/MM/YY | | | | 02.10.10 | | | To: DD/MM/YY | | | 01.10.11 | |
|  |  | | | | | | | | | | | |
|  | 2.3 Is this a student project? | | | | | | | | | | | |
|  | |  | |  | | YES |  | | NO | | | |
|  |  | | | | | | | | | | | |
|  | **DATA COLLECTION MUST NOT COMMENCE UNTIL ETHICS APPROVAL HAS BEEN GRANTED.** (National Statement, 2007, p.8) | | | | | | | | | | | |
|  |  | | | | | | | | | | | |
|  | [Note: *Multi-year approval may be given by the Committee. All projects, however, are subject to annual review. The Annual Renewal of projects is covered by the Progress/Final Report Form. Extensions beyond the approved duration are also covered by this form.*] | | | | | | | | | | | |
|  |  | | | | | | |  | | | | |
|  |  | | | | | | |  | | | | |
| **B.3** | **Is the research covered by a funding contract, agreement, or conditions of award?** *(please tick)* | | | | | | | | | | | |
|  |  | | Yes | | | | |  | | No | | |
|  |  | | Name of Funding Body: | | | | | | | | | |
|  |  | | Name of Grant: | | | | | | | | | |
|  |  | | Will the funding body have access to personally identifying information about the participants in the research?. | | | | | | | | | |
|  |  | | Yes | | | | |  | | No | | |
|  |  | |  | | | | | | | | | |
|  |  | |  | | | | |  | | | | |

| **SECTION C: RESEARCH DESIGN AND PROCEDURES** | | | | | | | | | |
| --- | --- | --- | --- | --- | --- | --- | --- | --- | --- |
|  |  |  |  | | |  | | |  |
| **C.1** | Brief description of project. | National Statement, 2007, s.1.2 | | | | | | | |
|  | *Please provide a brief description of the research proposal. Also, please attach to this application, in electronic format, a more detailed outline of the research design, objectives and methodology (2-4 pages*). | | | | | | | | |
|  | Metabolic syndrome is classified as a chronic, non-communicable disease, characterised by a combination of medical disorders that increase the risk of developing cardiovascular disease and type 2 diabetes. Several studies have investigated the effects of exercise on underlying risk factors leading to the development of the metabolic syndrome in older populations. However, limited studies have investigated the early risk factors for metabolic syndrome in young women. Therefore, this project aims to assess the influence and sustainability of a 12-week, multidiciplinary lifestyle intervention (physical activity, nutrition education, cognitive behavioural therapy) on risk factors for the metabolic syndrome, other cardiovascular health measures, and biochemical/immunological parameters. | | | | | | | | |
|  |  |  |  | | |  | | |  |
|  |  |  |  | | |  | | |  |
| **C.2** | Potential benefits of the research project. National Statement, 2007, s.1.1 | | | | | | | | |
|  | 1. To the participant:   Participants will be informed of their resting blood pressure, body composition, fasting plasma glucose, triglyceride levels, and cholesterol levels. In addition, this study will:  (1) Direct those young women potentially at risk of metabolic syndrome to local health care providers  (2) Provide the opportunity for improved cardiovascular health outcomes through participation in a lifestyle intervention . The "wait list" control nature of the study will ensure that all participants have access to the intervention. | | | | | | | | |
|  |  | | | | | | | | |
|  | 1. In general:   It is hoped that the results of this proposed study will:  (1) Advance the understanding of early signs of the metabolic syndrome in young women  (2) Further establish the understanding of the exercise, diet and psychosocial responses in young women who are at risk of developing the metabolic syndome  (3) Contribute to the advancement of young women’s health  (4) Lead to improved prescription of sustainable lifestyle intervention that influences psychosocial health and a reduction in the potential development of the metabolic syndrome | | | | | | | | |
|  |  |  |  | | |  | | |  |
| **C.3** | Brief description of the procedures to be followed. | | | | | | | | |
|  | *List sequentially the procedures which will apply to the participants, e.g., use of questionnaires, focus groups, interviews, and indicate any procedure/s which may have adverse effects.*  1.Initial contact with potential participants will occur by the researcher contacting health providers in local government areas  2. Participants will be provided with verbal and written details (refer to Information Letter to Participants) about all experimental procedures  3. Participants will attend their local GP for a general health check-up to ensure they are appropriate for participation. At this visit, they will have the GP complete the Cardiovascular Risk Assessment Form (find attached)  4. The following three documents will be completed once participants have been deemed suitable: Consent form, Young Women's Heart Health Study Survey, Activity and Food Record (find attached)  5. Supplying a 15 ml urine sample of the first void (urination) of the morning for assessment of microalbuminuria  6. Measurements of systolic and diastolic blood pressure  7. Anthropometric measurements of height, body mass, waist and hip circumference, and additional girth measures (i.e. relaxed & flexed bicep, mid-thigh, mid-calf)  8. Supplying fasting capillary blood samples (from an aseptic finger prick) for analysis of plasma glucose, triglyceride levels, HDL-cholesterol, and total cholesterol (requiring fasting from all food and beverage products, except water, for 12 hours prior)  9. Supplying a 10 ml intravenous blood sample, collected by a qualified phlebotomist, for assessment of cardiovascular risk factors (pro-thrombotic state, inflammatory markers, adipose tissue biomarkers)  10. Capturing echocardiogram images of the heart for standard analysis of cardiac structure and function using non-invasive B-mode ultrasonography. This will require simultaneous ECG tracing. This process is expected to take 20-30 minutes. Following the analysis, a software program will be employed to analyse the images off-line.  11. Determination of vascular endothelial function via flow-mediated dilation using non-invasive B-mode ultrasonography. Brachial arterial diameter and blood flow velocity will be calculated at baseline (resting), during reactive hyperaemia (after the occlusion of the forearm at 250 mmHg for 4.5 mins), and after sublingual nitroglycerine ingestion (400 ug - doses explained in attachments1/2)  12. Non-invasive ultrasound measures at the carotid artery for assessment of intima-media thickness  13. Submaximal exercise test (on a cycle ergometer) with simultaneous recording of heart rate response and ratings of perceived exertion at one minute intervals  14. The assessment of upper and lower body strength (using a combination of free and machine weights commonly prescribed) for the determination of five-repetition max  15. Participate in a 12-week aerobic exercise and resistance training intervention (3 days of structured exercise per week)  16. Keeping a training diary (find attached)  17. Participate in weekly focus groups, lasting one hour, about behavioural change delivered by a qualified counsellor  18. Baseline and weekly nutritional education directed by a dietician  19. The completion of five psychological inventories about personal beliefs and attitudes towards exercise (find attached) | | | | | | | | |
|  |  | | | | | | | | |
|  |  | | | | | | | | |
| **C.4** | Risks to participants. National Statement, 2007, s.2.1 | | | | | | | | |
|  | If there are any risks to participants, please describe the risks and the measures that will be taken to limit them*.* For participant safety, individuals will be excluded if they have a history of: heart disease, heart arrhythmias, respiratory condition, are pregnant or breast feeding or taking any contraindicatory medications. In the unlikely event that an adverse reaction occurs during the exercise intervention, the participants will be advised to stop immediately and undergo furthre monitoring. | | | | | | | | |
|  |  | | | | | | | | |
|  | If there is any chance that the participants may become distressed, alarmed or disadvantaged in any way, please identify a person to whom they may be referred for counselling or other appropriate support. [Note: *It is normally not appropriate for investigators, supervisors or student researchers to undertake this role themselves*.]  If participants become psychologically distressed, alarmed, or disadvantaged while participating in the study, it is recommended that they call Lifeline Melbourne on 13 11 14, which is a 24 hour telephone counselling service for people over 18 years. Physical events will be referred to their general practitioner. | | | | | | | | |
|  |  | | | | | | | | |
|  |  | | | | | | | | |
| **C.5** | Administration of drugs, compounds, or biological agents. | | | | | | | | |
|  | Does your research involve the administration of any substance or agent? | | |  | YES | |  | NO | |
|  | If “YES”, please complete Attachment 1 and append it to your application. | | | | | | | | |
|  |  | | |  |  | |  |  | |
|  |  | | |  |  | |  |  | |
| **C.6** | Use of body tissues or fluids | | |  |  | |  |  | |
|  |  | | |  |  | |  |  | |
|  | Does your research involve any procedures to remove body fluids or tissues? | | |  | YES | |  | NO | |
|  | If “YES”, please complete Attachment 2 and append it to your application | | | | | | | | |
|  |  | | |  |  | |  |  | |

| **SECTION D: PROJECT PARTICULARS** | | | | | | | | | | | | | | |
| --- | --- | --- | --- | --- | --- | --- | --- | --- | --- | --- | --- | --- | --- | --- |
|  |  | | | | | | | | | | | | |  |
| D.1 | Participant Details. National Statement, 2007, s.1.4 | | | | | | | | | | | | |  |
|  |  | | | | | | | | | | | | |  |
| **D.1.1** | Brief description of participants: | | | | | | | | | | | | |  |
|  | [E.g., Year 11 students in public schools, childless couples who have been married for 10 or more years, nurses who have been working for at least 5 years etc.] Participants will have the following characteristics:  * Female  * Raised waist circumference (>80 cm)  * Have not been involved in regular exercise in the six months prior to data collection | | | | | | | | | | | | |  |
|  |  | | | | | | | | | | | | |  |
| **D.1.2** | Estimated number involved: | | | Females | | 124 | | | Males | 0 | Total | | 124 |  |
|  |  | | | | | | | | | | | | |  |
| **D.1.3** | Age range for each gender: | | | Females | | 18-30 | | | Males | N/A | |  | |  |
|  |  | | |  | | | | | | | | | |  |
| **D.1.4** | Participants’ state of health | | |  | Normal | | | | | | | | |  |
|  |  | | |  | Other (please specify) | | | | | | | | |  |
|  |  | | | | participants will have a raised waist circumference and the possibility of other metabolic syndrome risk factors | | | | | | | | |  |
|  |  | | | |  | | | | | | | | |  |
| **D.1.5** | Method of recruitment of participants (including how participants will be approached) | | | | | | | | | | | | |  |
|  | [Note: Researchers who intend to use their own students, patients, clients etc. as participants need to be especially aware of the potential risks, e.g., coercion, misuse of power.] | | | | | | | | | | | | |  |
|  | Participation is voluntary.  Participants will be recruited via local health promotion networks including GPs and social workers. A letter of invitation for participation will be distributed. | | | | | | | | | | | | |  |
|  |  | | | | | | | | | | | | |  |
| **D.1.6** | **Conflicts of Interest.** National Statement, 2007, ch.5.4 and ss.5.2.10 and 5.2.11 | | | | | | | | | | | | |  |
| **D.1.6.1** | Researchers who intend to use their own students, patients, clients, etc. as  participants need to be aware of the potential risk of conflicts of interest, for example, of coercion, misuse of power, etc.   Does your research involve possible conflicts of interest in relation to  recruitment and use of participants? | | | | | | | | | | | | |  |
|  |  | | | | | | | | | | | | |  |
|  |  |  | YES | | | |  | NO | | | | | |  |
|  | If “YES”, describe how you will deal with this. | | | | | | | | | | | | |  |
|  |  | | | | | | | | | | | | |  |
|  |  | | | | | | | | | | | | |  |
| **D.1.6.2** | Other conflicts of interest may arise in relation to research undertaken in one’s place of employment or through financial or other interest or affiliation that bears on the research. Does your research involve any conflicts of interest? | | | | | | | | | | | | |  |
|  |  |  |  | | | |  |  | | | | | |  |
|  |  |  | YES | | | |  | NO | | | | | |  |
|  | If “YES”, describe the conflicts of interest and how they are being dealt with. | | | | | | | | | | | | |  |
|  |  | | | | | | | | | | | | |  |
|  |  | | | | | | | | | | | | |  |
| **D.1.7** | **Compensation to participants.** National Statement, 2007, s.2.2.9 | | | | | | | | | | | | |  |
|  | Will a reward or incentive of any kind be offered to the participants? | | | | | | | | | | | | |  |
|  |  |  |  | | | |  |  | | | | | |  |
|  |  |  | YES | | | |  | NO | | | | | |  |
|  | If “YES”, provide full details. | | | | | | | | | | | | |  |
|  | Participants will be able to access information regarding their personal health status obtained from our assessment. Furthermore, each participant will receive a token of appreciation for their commitment to the study (for example, travel reimbursement). | | | | | | | | | | | | |  |
|  |  | | | | | | | | | | | | |  |

| **D.1.8** | **Involvement of special groups of participants** | | | | | |
| --- | --- | --- | --- | --- | --- | --- |
|  |  | | | | | |
| **D.1.8.1** | Will participants be selected specifically based on cultural or community groups to which they belong? (e.g., Aboriginal or Torres Strait Islander peoples, Asian communities)? | | | | | |
|  |  |  |  | |  |  |
|  |  |  | YES | |  | NO |
|  |  |  |  | |  |  |
|  | In the case of research involving Indigenous issues or people: (a) the [Values and Ethics - Guidelines for Ethical Conduct in Aboriginal and Torres Strait Islander Health Research](http://www7.health.gov.au/nhmrc/publications/synopses/e52syn.htm) must be adhered to; and (b) the application must be forwarded to a Head of one of the University’s three Indigenous Support Units for comment prior to submission to the Research Office and (c) written comment from the Head, ATSI unit must be attached to this application. | | | | | |
|  |  | | | | | |
| **D.1.8.2** | If yes, do these participants require permission from community leaders? | | | | | |
|  |  |  |  | |  |  |
|  |  |  | YES | |  | NO |
|  |  |  |  | |  |  |
| **D.1.9** | Does your research involve participants in other countries? If so, please advise the committee as required under National Statement, 2007, s.4.8.4 of information relating to any ethical processes or approval required in that country. Please attach any relevant documentation. | | | | | |
|  |  | | | | | |
|  |  | | | | | |
|  |  | | | | | |
| D.2 | **Access to personal information, data/files/records or samples of human tissue** | | | | | |
|  |  | | | | | |
|  | Will the project involve access to personal information, student files, computerised records or other data banks, human pathology or diagnostic specimens provided by one or more institutions or government departments? | | | | | |
|  |  | | | | | |
|  |  |  | YES | |  | NO |
|  |  | | | | | |
|  | If “YES”, please identify the sources and location of the data. | | | | | |
|  |  | | | | | |
|  |  | | | | | |
|  | Again, if “YES”, will the identity of the participants be known in any way? | | | | | |
|  |  | | | | | |
|  |  |  | YES | |  | NO |
|  |  | | | | | |
|  | Please explain how they will be known and what will be done with the data. | | | | | |
|  |  | | | | | |
|  |  | | | | | |
|  |  | | | | | |
| D.3 | **Location of Study** | | | | | |
|  |  | | | | | |
|  | **Where will the research be conducted?** | | | | | |
|  |  | | | | | |
| **D.3.1** | **If outside ACU, give name and address of institution and contact names:** | | | | | |
|  | Ideally all testing will be performed at ACU. However, where necessary, some of the biochemical health parameters may be assessed off campus. In this instance, venue will be advised.  Furthermore, some of the exercise sessions may be performed off-campus for variety or participant convenience. | | | | | |
|  |  | | | | | |
| **D.3.2** | **If at ACU, give campus location:** | | | School of Exercise Science  Melbourne (St. Patrick's Campus) | | |
|  |  | | | | | |
|  |  | | | | | |

| D.4 | | **Approval from institutions or organisations external to ACU to access participants**. National Statement, 2007, s.2.2.13 | | | | | | | |  |
| --- | --- | --- | --- | --- | --- | --- | --- | --- | --- | --- |
|  | | *[Note: Researchers should be aware of the requirements set out in the current privacy legislation.]* | | | | | | | |  |
|  | |  | | | | | | | |  |
| **D.4.1** | | **Is formal approval required to access participants from an external institution or organisation?** | | | | | | | |  |
|  | | *(E.g., from the state Department of Education, Catholic Education Office, School Principal/s, Hospital HREC)*  [Note: *If participants are to be recruited from schools, hospitals, prisons or other institutions, approval from the institution or appropriate authority must be sought.*] | | | | | | | |  |
|  | |  | | | | | | | |  |
|  | |  | |  | | YES |  | NO | |  |
|  | |  | | | | | | | |  |
|  | |  | | | | | | | |  |
| **D.4.2** | | **Please indicate whether formal approval has already been obtained from the appropriate authorities of other institution(s) or from another HREC:** | | | | | | | |  |
|  | | [*Please refer to Part B, Section 2.2 and Section 3 of the Guidelines*.] | | | | | | | |  |
|  | |  |  | | YES – If “Yes”, when was it obtained? | | | |  |  |
|  | |  | | | Please attach a copy of the formal clearance/permission | | | | |  |
|  | |  | | | | | | | |  |
|  | |  |  | | NO | | | |  |  |
|  | |  | | | Please attach a copy of the letter of request or of the Application Form used by the relevant institutions or organisations, or please indicate when such approval will be requested:  N/A | | | | |  |
|  | |  | | | | | | | |  |
|  | |  | | | | | | | |  |
| **D.5** | | **Informed consent of participants, parents or guardians of minors, next-of-kin, community leaders** | | | | | | | |  |
|  | |  | | | | | | | |  |
| **D.5.1** | | **Will persons aged 18 or above be asked to complete a Consent Form?** National Statement, 2007, Ch 4.2 | | | | | | | |  |
|  | |  | | | | | | | |  |
|  | |  |  | | YES (If “Yes”, attach a copy of the proforma to this application. See sample Consent Form at www.acu.edu.au/research) | | | | |  |
|  | |  |  | |  | | | | |  |
|  | |  |  | | NO (If “No”, please explain; e.g., participants will be completely anonymous at every stage of the project.) | | | | |  |
|  | |  |  | |  | | | | |  |
|  | |  | | | | | | | |  |
| **D.5.2** | | **Consent of parents/guardians to access minors** | | | | | | | |  |
|  | |  | | | | | | | |  |
|  | | [Note: *See Guidelines Part B, Sections 4.8 and 6.2. National Statement, 2007, Ch.4.2.*] | | | | | | | |  |
|  | |  | | | | | | | |  |
|  | | Does the research involve minors as participants and therefore require the consent of parents/guardians? | | | | | | | |  |
|  | |  | | | | | | | |  |
|  | |  | |  | | YES |  | NO | |  |
|  | |  | | | | | | | |  |
|  | | “If "Yes", a copy of the proforma for gaining the consent of the primary care-giver must be attached to this Application. See the sample Parent/Guardian Consent Form at www.acu.edu.au/research | | | | | | | |  |
|  | |  | | | | | | | |  |
| **D.5.3** | | **Consent of person responsible for those unable to give consent.** National Statement, 2007, Ch 4.3, 4.4, 4.5 | | | | | | | |  |
|  | |  | | | | | | | |  |
|  | | [Note: *See also Guidelines Part B, Section 6.2.*] | | | | | | | |  |
|  | |  | | | | | | | |  |
|  | | Does the research involve participants who are unable to give consent (e.g., because they have an intellectual or mental impairment, or because they are highly dependent on medical care)? | | | | | | | |  |
|  | |  | | | | | | | |  |
|  | |  | |  | | YES |  | NO | |  |
|  | |  | | | | | | | |  |
|  | | If "Yes", a copy of the proforma for gaining the consent of the person responsible must be attached to this Application. | | | | | | | |  |
|  | |  | | | | | | | |  |
| **D.5.4** | | **Consent of community leaders** | | | | | | | |  |
|  | |  | | | | | | | |  |
|  | | Does the research involve participants from special groups or communities where such approvals are customary? | | | | | | | |  |
|  | |  | | | | | | | |  |
|  | |  | |  | | YES |  | NO | |  |
|  | |  | | | | | | | |  |
|  | | If “Yes”, a copy of the proforma for gaining the consent of the community leader must be attached to this application. | | | | | | | |  |
|  | |  | | | | | | | |  |
| **SECTION E: GATHERING OF DATA, SECURITY OF DATA, DISPOSAL OF DATA, AND DISSEMINATION OF RESULTS** | | | | | | | | | | |
| See Chapter 2, Australian Code for the Responsible Conduct of Research | | | | | | | | | | |
| **E.1** | **Gathering of data** | | | | | | | | | |
|  |  | | | | | | | | | |
| **E.1.1** | How will the data be gathered? | | | | | | | | | |
|  | Health screening data (questionnaires, surveys, and psychological inventories) will be recorded in hard copy. Blood samples required for the determination of cholesterol, triglycerides and fasting plasma glucose will be collected by aseptic finger-prick technique using a safety lancet and collected into capillary tubes. A small droplet of this blood is placed onto measuring strips and fed into the (Reflotron Plus) blood analyser. Intravenous blood samples will be collected by a qualified phlebotomist and sent to a local pathology clinic for subsequent determination of cardiovascular risk factors, including pro-thrombotic state, pro-inflammatory levels and adipose tissue biomarkers. The first void (urination) of the morning will be collected into urine containers provided in advance to each participant. Microalbuminuria will be determined from the 15 ml urine sample using a specialised analysis kit. Girths and circumference measurements will be established using a measuring tape, to the nearest 0.5 cm. Body mass will be recorded with electronic scales; height willl be established using a stadiometer. Systolic and diastolic blood pressure will be measured using an electronic sphygmomanometer. Collection of these measures will take place in a secure and private room by a female investigator. For the analysis of cardiac structure and function, echocardiogram images of the heart will be captured using the portable Vivid I cardiovascular ultrasound device. During the analysis, participants will be simultaneously connected to a 3-lead ECG. Assessment of vascular endothelial function requires a pneumatic cuff to be placed on the participant's forearm near the elbow, and inflated to 250 mmHg for 4.5 minutes (Jarvisalo, et al., 2004). Upon release, a doppler probe will be used to analyse flow mediated dilation at the brachial artery. Flow mediated dilation will also be assessed following sublingual adminstration of nitroglycerin. Furthermore, ultrasound techniques at the carotid artery, for the assessment of intima-media thickness, will take place whilst the participant is in a supine position. | | | | | | | | | |
|  |  | | | | | | | | | |
| **E.1.2** | How will the data be recorded? | | | | | | | | | |
|  | Results from hard copy surveys will be entered into an electronic data base. Where possible, results will be directly entered into a computer data spreadsheet for subsequent statistical analysis. | | | | | | | | | |
|  |  | | | | | | | | | |
|  |  | | | | | | | | | |
| **E.2** | **Security of Data**  Data needs to be regularly archived in a secure environment, in a room at ACU during the study, and held for a minimum of five years following completion of the study. | | | | | | | | | |
|  |  | | | | | | | | | |
|  | [Note: See Guidelines, Part B, Sections 8.1 and 8.2] | | | | | | | | | |
|  |  | | | | | | | | | |
| **E.2.1** | In which room and at which campus of ACU will the primary data be stored during the study? | | | | | | | | | |
|  | LG 49 (Principal supervisors office) and LG 64 (postgraduate research room) Melbourne, St. Patrick's campus | | | | | | | | | |
|  |  | | | | | | | | | |
| **E.2.2** | In which room and at which campus of ACU will the data be stored following completion of study? | | | | | | | | | |
|  | LG 49 - office of the Assistant Head of the School of Exercise Science, Dr Justin Kemp | | | | | | | | | |
|  |  | | | | | | | | | |
|  |  | | | | | | | | | |
| **E.3** | **Disposal of data** | | | | | | | | | |
|  |  | | | | | | | | | |
|  | How are the data to be disposed after complying with the requirement to retain data for a minimum of five years (e.g., erasing of tapes, shredding of questionnaires, deletion of electronic data)? | | | | | | | | | |
|  | All data will be collected in an electronic format and will be disposed of by deletion of files after 5yrs. All hard copy data will be shredded or disposed of into in-confidence bins. | | | | | | | | | |
|  |  | | | | | | | | | |
|  |  | | | | | | | | | |

| **E.4** | **Dissemination of results** | | | | |
| --- | --- | --- | --- | --- | --- |
|  |  | | | | |
|  | Do you intend to use the results of your study in publications or in other communications with colleagues? | | | | |
|  |  | | | | |
|  |  |  | YES |  | NO |
|  |  | | | | |
|  | If "Yes", participants must be advised both in the Information Letter to Participants and on the Consent Form, if applicable, that results from the study may be summarised and appear in publications or may be provided to other researchers in a form that does not identify the participants in any way. | | | | |
|  |  | | | | |

| **SECTION F: IDENTIFIABILITY OF PARTICIPANTS.** National Statement, 2007, Ch.3.2 | | |
| --- | --- | --- |
| **F.1** | In what format will the data be collected? | |
|  |  | |
|  |  | **Individually identifiable data** where the identity of a specific individual can reasonably be ascertained. Examples of identifiers include the individual’s name, image, date of birth or address; |
|  |  | **Re-identifiable data**, from which identifiers have been removed and replaced by a code, but it remains possible to re-identify a specific individual by, for example, using the code or linking different data sets; |
|  |  | **Non-identifiable data**, which have never been labelled with individual identifiers or from which identifiers have been permanently removed, and by means of which no specific individual can be identified. A subset of nonidentifiable data are those that can be linked with other data so it can be known that they are about the same data subject, although the person’s identity remains unknown. |
|  |  | |
| **F.2** | Please indicate whether the identity of any participant will be disclosed to anyone other than the researcher/s, and, if so, please explain the reasons for this disclosure. | |
|  | The identital of all participants will be replaced by a code. Only the research student, supervisor and co-supervisors will have access to the identity of participants. | |
|  |  | |

| **SECTION G: CONFIDENTIALITY OF PARTICIPANTS’ RESPONSES** | |
| --- | --- |
|  | |
| **Confidentiality** refers to the obligation pf people not to use private information – whether private because of its content or the context of its communication – for any purpose other than that for which it was given to them. (*National Statement*, 2007, p.99) The information given is to be used only for the research purposes stated in the protocol. Without the explicit permission of the person providing it, such information must not be divulged to others in any way that might allow it to be linked to that person. | |
|  |  |
| **G.1** | What measures will be taken to ensure the confidentiality of the personal information gathered in this project (e.g., removal of names and other identifiers either before, during or after analysis of data; reporting aggregated data only)? |
|  | Descriptive statistics of the group will only be reported in publications. All spread sheets used for the study will have participants' ID and not their names |
|  |  |
| **G.2** | In this project are there any particular risks to the confidentiality of personal information (e.g., reporting non-aggregated data or descriptive data from small samples)? If so, how is it proposed to minimise them? |
|  | None |
|  |  |

| **SECTION H: INFORMATION PRIVACY** | | | | | |
| --- | --- | --- | --- | --- | --- |
|  |  | | | | |
| Researchers should be familiar with the existence of relevant Commonwealth, State and Territory legislation regarding privacy. Of special note are the *Information Privacy Principles - IPPs* (see Appendix D to the *Guidelines*) and the *National Privacy Principles – NPPs* (from the *Privacy Act* 1988 (Commonwealth), incorporating the *Privacy Amendment* (Private Sector) *Act* 2000 (Commonwealth)). | | | | | |
|  |  | | | | |
| **H.1** | Are you aware of any privacy issues that may impact on participants? | | | | |
|  |  | | | | |
|  |  |  | YES |  | NO |
|  |  | | | | |
|  | If “Yes” please identify this issue (making reference to IPPs and NPPs). | | | | |
|  |  | | | | |
| **H.2** | If applicable, please identify the IPPs/NPPs which are being cited to justify the use of identifiable data without seeking the participant’s consent. | | | | |

| **SECTION I: ETHICAL ISSUES – still to be addressed** | |
| --- | --- |
|  |  |
| **If you answered “YES” to any of the areas on the checklist in Section A (above) and have not commented on those areas in any other section of this Application Form, please indicate here how you intend to address the ethical issues arising.** | |
|  | The areas in Section A to which "Yes" was answered have each been addressed in the detailed outline of the research design, objectives and methodology (see C.1 above and attachment 3). |
|  |  |

| **SECTION J: CHECK LIST – to be completed before submitting the application** | | | | | |
| --- | --- | --- | --- | --- | --- |
| Please tick, as appropriate | | | **YES** | **NO** | **N/A** |
| **J.1** | The Guidelines have been read and adhered to. | |  |  |  |
| **J.2** | All sections of the application form have been completed. | |  |  |  |
|  |  | |  |  |  |
| **J.3** | Details of participant requirements have been fully described. | |  |  |  |
|  |  | |  |  |  |
| **J.4** | All relevant supporting documents are attached: | |  |  |  |
|  |  |  |  |  |  |
|  | 4.1 | Documentation from the Indigenous Support unit. |  |  |  |
|  |  |  |  |  |  |
|  | 4.2 | Copies of any external approval forms to be submitted to hospitals, schools, etc |  |  |  |
|  |  |  |  |  |  |
|  | 4.3 | If your research involves an external organisation, the letter from the organisation agreeing to be involved in the research is attached. |  |  |  |
|  |  |  |  |  |  |
|  | 4.4 | Ethics approval from external institutions (e.g., hospitals, schools) if available. |  |  |  |
|  |  |  |  |  |  |
|  | 4.5 | Research Proposal (as requested at Section C.1 of the Application Form) |  |  |  |
|  |  |  |  |  |  |
|  | 4.6 | Information Letter to Participants and consent forms are on University Letterhead. |  |  |  |
|  |  |  |  |  |  |
|  | 4.7 | Information Letter to Participants and consent forms follows the recommended format and wording as at[www.acu.edu.au/research](http://www.acu.edu.au/research) and are in plain English |  |  |  |
|  |  |  |  |  |  |
|  | 4.8 | Two copies of the Consent Form have been provided. |  |  |  |
|  |  |  |  |  |  |
|  | 4.9 | Copies of all questionnaires and interview schedules. (If interviews are to be open-ended, a list of sample questions for each stage of the interview schedule.) |  |  |  |
|  |  |  |  |  |  |
|  | 4.10 | In cases of more than low risk to participants (at Section A.6 of the Application Form), copy of statement from medical practitioner, psychologist, counsellor prepared to provide professional assistance as required for procedures which might have an adverse effect on a participant’s well-being. |  |  |  |
|  |  | |  |  |  |
| **J.5** | **The Information Letters and this application have been checked for typographical, spelling and grammatical errors.** | |  |  |  |

| **SECTION K: DECLARATION –** to be completed before submitting the application electronically. |
| --- |

I/We declare that the information I/We have given above is true and correct in all respects and that I/We have disclosed all aspects of the project. I am/We are familiar with and have access to copies of the National Health and Medical Research Council’s *National Statement on Ethical Conduct in Human Research* (2007).

I/We accept responsibility for the conduct of this research in accordance with the principles contained in the NHMRC Statement and any other conditions specified by the Human Research Ethics Committee of Australian Catholic University.

I/We will notify the Human Research Ethics Committee immediately of any variation to this project, e.g., changes to the number or mix of participants, to research procedures, to the survey instrument(s) or questionnaire(s).

**You are reminded that contact with participants and/or access to their records/files/specimens must not commence until written ethics approval has been received from the HREC.**

**I/We declare that we will NOT commence data collection and/or access participants’ records/files/specimens until written approval has been received from the Human Research Ethics Committee.**

| **Name (block letters)** | **Signature** | **Date** |
| --- | --- | --- |
|  |  |  |
|  |  |  |
| Principal Investigator or Supervisor |  |  |
|  |  |  |
|  |  |  |
|  |  |  |
| Co-Investigator 1 or Co-Supervisor (if staff) or Student Researcher 1 (if applicable) | |  |
|  |  |  |
|  |  |  |
|  |  |  |
| Co-Investigator 2 (if staff) or Student Researcher 2 (if applicable) | |  |
|  |  |  |
|  |  |  |
|  |  |  |
| Co-Investigator 3 (if staff) or Student Researcher 3 (if applicable) | | |
|  |  |  |
|  |  |  |
|  |  |  |
| Co-Investigator 4 (if staff) or Student Researcher 4 (if applicable) | |  |
|  |  |  |
|  |  |  |
|  |  |  |
| Co-Investigator 5 (if staff) or Student Researcher 5 (if applicable) | |  |

| **PRIVACY STATEMENT:**  Australian Catholic University is committed to ensuring the privacy of all information it collects. Personal information supplied to the University will only be used for administrative and educational purposes of the institution. Personal information collected by the University will only be disclosed to third parties with the written consent of the person concerned, unless otherwise prescribed by law. For further information, please see the University’s Statement on Privacy http://www.acu.edu.au/privacy_policy.cfm. |
| --- |

| **ATTACHMENT 1** |
| --- |

AUSTRALIAN CATHOLIC UNIVERSITY

Human Research Ethics Committee

|  |  |
| --- | --- |
|  | **Administration of Substances/Agents**  *Detailed information on any chemical compounds, drugs or biological agents is required, together with indications of dosage, frequency of administration and anticipated effects.* |
|  |
|  |
|  |
|  | - Name(s) of Substance(s):  Nitroglycerine |
|  |
|  |
|  | - Dosage per administration:  400 micrograms (Corretti et al., 2002; Walther et al., 2008) |
|  |
|  |
|  | - Frequency of administration:  Administered sublingually once per testing session. There will be three testing sessions in total, each separated by three months. |
|  |
|  |
|  | - Total amounts to be administered:  1200 micrograms |
|  |
|  |
|  | - Anticipated effects:  Involuntary vasodilatory response |
|  |
|  |
|  | - Other comments to assist the Committee:  The use of nitroglycerin is a well-established protocol previously used in cardiac research with paediatric, adolescent and adult populations (Corretti et al., 2002) |
|  |
|  |

| **PRIVACY STATEMENT:**  Australian Catholic University is committed to ensuring the privacy of all information it collects. Personal information supplied to the University will only be used for administrative and educational purposes of the institution. Personal information collected by the University will only be disclosed to third parties with the written consent of the person concerned, unless otherwise prescribed by law. For further information, please see the University’s Statement on Privacy http://www.acu.edu.au/privacy_policy.cfm. |
| --- |

| **ATTACHMENT 2** |
| --- |

AUSTRALIAN CATHOLIC UNIVERSITY

Human Research Ethics Committee

|  |  |
| --- | --- |
|  | **Sampling of Body Tissue or Fluids** |
|  |  |
|  | *If the research involves administration of foreign substances or invasive procedures, please attach a statement from a medical or paramedical practitioner with indemnity insurance, accepting responsibility for those procedures.* |
|  |
|  | - What will be sampled and how?  (a) Blood samples will be collected from each participant for lipid profiling of cholesterol and triglycerides, and plasma glucose concentration. The Reflotron Plus, a portable blood analysis device, will be employed. Blood is collected by the aseptic finger-prick technique using a safety lancet and collected into capillary tubes. A droplet of blood (30 ul) is placed onto measuring strips and placed in the Reflotron Plus analyser.  (b) Intravenous blood samples will be collected by the research student (accredited phlebotomist) and sent to a pathology clinic for analysis of cardiovascular risk factors .  (c) Urine from the first void (urination) of the morning will be provided by participants in a urine container; they will be asked to supply a mid-flow urine sample upon waking on the day of testing |
|  |
|  |
|  | - Frequency and volume?  All procedures below will be performed at the three testing sessions of the study (with sessions separated by 12 weeks):  1. Capillary blood samples (150 ul) will be collected from the fingertip.  2. A 10 ml intraveous blood sample will be collected from the antecubital vein.  3. A 15 ml urine sample will be collected. |
|  |
|  |
|  | - How are samples to be stored?  Capillary blood samples will be immediately analysed and disposed of in Biohazard waste bins  All intravenous blood samples will be stored according to common practices and sent to a local pathology clinic  Urine samples will be immediately analysed and disposed of, requiring no need for storage |
|  |
|  |
|  | - How will samples be disposed of?  Blood samples, capillary tubes and urine samples will be disposed of in appropriate Biohazard bins immediately after analysis |
|  |
|  |
|  | - Who will take the samples?  Individuals trained in blood collection (see below) |
|  |
|  |
|  | What are their qualifications for doing so?  Trained in the collection of blood samples - specifically, IV canulation and venepuncture (A&A Training and Consultancy).  Previous experience in the collection of finger-prick blood samples through B.Ex.Sc, B.Ex.Sc (Honours), and employment history.  Urine is collected by the participant. |
|  |
|  |
|  | - Other comments to assist the Committee:  Further details are provided in the summary (attachment 3) |
|  |
|  |

| **PRIVACY STATEMENT:**  Australian Catholic University is committed to ensuring the privacy of all information it collects. Personal information supplied to the University will only be used for administrative and educational purposes of the institution. Personal information collected by the University will only be disclosed to third parties with the written consent of the person concerned, unless otherwise prescribed by law. For further information, please see the University’s Statement on Privacy http://www.acu.edu.au/privacy_policy.cfm. |
| --- |
