# Supplementary material for: Effects of a Multi-Disciplinary Lifestyle Intervention on Cardiometabolic Risk Factors in Young Women with Abdominal Obesity: A Randomised Controlled Trial
Source: PLoS One. 2015 Jun 26;10(6):e0130270. doi: 10.1371/journal.pone.0130270 (PMC4483260; doi:10.1371/journal.pone.0130270)
Supplement: S2 Text — (DOC) [file pone.0130270.s004.doc]

**Background**

The metabolic syndrome is classified as a chronic, non-communicable disease, characterised by a combination of medical disorders that increase the risk of developing cardiovascular disease and type 2 diabetes (Alberti, Zimmet, & Shaw, 2006). The incidence of the metabolic syndrome is rising rapidly, becoming a health care crisis of epidemic proportion faced by the western world (World Health Organisation [WHO], 2008). Incidences of the metabolic syndrome may be as high as 29 per cent in Australian adults aged 25 years and over (Cameron, Magliano, Zimmet, Welborn, & Shaw, 2007). Those individuals with the metabolic syndrome are twice as likely to die from cardiovascular disease and have a fivefold greater risk of developing type 2 diabetes (Zimmet, Alberti, & Shaw, 2005). The underlying causative factors of the metabolic syndrome are acknowledged to be both insulin resistance and abdominal obesity (Barr et al., 2006). Environmental factors such as those associated with a sedentary lifestyle are also responsible for its development (IDF, 2006).

**Rationale**

Both gender and low socioeconomic status are key determinants of health inequality (AIHW, 2008) and may be linked to relatively high risks of associated long-term health conditions, particularly cardiovascular disease and type 2 diabetes. To date, most research into the metabolic syndrome has been conducted in people aged over 40 years, even though earlier detection may be the best public health approach (Galassi, Reynolds, & He, 2006). Furthermore, the effectiveness of lifestyle intervention programs on risk factors of the metabolic syndrome in young women is poorly understood. Therefore, this study aims to determine the influence of a lifestyle intervention in young women from areas of socioeconomic disadvantage and provide evidence for future preventative strategies.

**Objectives**

This project aims to assess the influence and sustainability of a 12-week lifestyle intervention on risk factors of the metabolic syndrome (waist circumferences, blood pressure, triglycerides, fasting plasma glucose, and HDL-cholesterol), other cardiovascular and biochemical/immunological health parameters, and psychosocial health in young Caucasian women aged 18 to 30 years and residing in socioeconomically disadvantaged areas of urban Victoria. The outcomes of this work will advance the understanding of the metabolic syndrome in young women.

**Methodology**

Participants

| **Inclusion Criteria** | **Exclusion Criteria** |
| --- | --- |
| - Female | - Previously diagnosed: polycystic ovarian syndrome, thyroid abnormalities, insulin-dependent diabetes mellitus, gestational diabetes, cardiovascular disease, heart arrhythmia, liver or kidney disease, or respiratory conditions |
| - 18 – 30 years | - Use of medications that could confound exercise response |
| - Abdominal obesity | - Pregnant or breast feeding |
| - Sedentary lifestyle behaviour (do not participation in regular physical activity) |  |
| - Caucasian |  |
| - Reside in a socioeconomically disadvantaged suburb of Victoria (SEIFA Index) |  |

Study design

One hundred and twenty four young women identified as being at risk of developing the metabolic syndrome, as defined by the International Diabetes Federation (2006), will be invited to participate in a 12-week, randomised controlled lifestyle intervention. Sixty two women will act as the experimental intervention group, whilst the remaining women will act as a “wait list” control group. This study design ensures that all women will have access to the intervention. There will also be a sustainability component included in this study, whereby all participants are followed up three months after the conclusion of the lifestyle intervention to assess physiological markers of the metabolic syndrome and changes in psychosocial status. The Lifestyle ntervention will include the following:

1. *Physical activity*

A progressive circuit training program comprising resistance and aerobic exercise will be employed during the exercise component of the intervention. The exercise component will include training three days per week. The two-plus-one model will be implemented, whereby participants will engage in two training sessions supervised by the research student, complemented with one session of unsupervised training (i.e. location and time of their choice). Participants will wear a heart rate monitor and a pedometer to monitor training intensity.

1. *Nutrition education*

Participants will be supplied with nutritional advice sourced from a dietician on a regular basis regarding issues around healthy food and lifestyle education

1. *Cognitive behavioural therapy*

Participants will partake in weekly focus group sessions for educational coaching about cognitive behavioural change, delivered by a qualified counsellor. Participants will complete five psychological inventories at baseline, post-intervention, and 3-months post-intervention to establish their personal beliefs and attitudes towards exercise.

Study data

Data collection will occur at three time points: (1) pre-intervention; (2) post-intervention; (3) 12-weeks post-intervention (sustainability component). Each session will be carried out by the research student and involve the following testing procedures:

*Metabolic syndrome risk factors*

Assays for fasting plasma glucose concentration, serum triglycerides, total cholesterol and HDL-cholesterol will be obtained from an aseptic finger prick, collected into capillary tubes, and analysed using the Reflotron Plus™ analyser. This will require abstaining from all food and beverage products, except water, for a minimum of 12 hours prior to testing. Participants will also be required to refrain from consuming alcohol and the participation in strenuous physical activity in the 24 hours prior to testing. Resting systolic and diastolic blood pressure measurements will be assessed after each participant has been seated quietly for 20 minutes.

*Anthropometry measures*

Stature (standing height), body mass, and waist and hip circumference will be measured. Additional girths (i.e. bicep relaxed, bicep flexed, mid-thigh, calf) will also be collected. All measurements will be performed by a female researcher in a private room.

*Fitness and strength assessment*

Participants will undergo a graded, submaximal cycle ergometer stress test for the assessment of cardiovascular fitness (lasting up to 12 minutes). Additionally, upper (chest press) and lower body (leg press) strength will be calculated via a five-repetition maximal resistance test.

*Other biochemical/ immunological measures*

Participants will be asked to supply a 10 ml intravenous blood sample for assessment of cardiovascular risk factors (i.e. pro-thrombotic state, pro-inflammatory indicators, and adipose tissue biomarkers). All samples will be appropriately stored according to common practices and sent to a local pathology clinic for analysis. Blood samples will be collected by an individual trained in the collection of intravenous blood.

*Cardiac measures*

Capturing echocardiogram images of the heart for standard analysis of cardiac structure and function will be performed by the research student using non-invasive B-mode ultrasonography. This will also require simultaneous ECG tracing. Following the analysis, a software program will be employed to analyse the images off-line.

*Vascular measures*

Markers for vascular endothelial function will be assessed via flow-mediated dilation using non-invasive B-mode ultrasonography. A measure of resting diameter and blood flow velocity at the brachial artery will be recorded. The forearm will then be occluded with a pneumatic tourniquet at 250 mmHg for 4.5 minutes. Arterial diameter and blood flow velocity will be re-measured upon cuff release. A small sublingual dose (400 $\mu$g) of nitroglycerin will then be administered to induce arterial dilatation. Following this, the aforementioned measures (without forearm occlusion) will again be taken at the brachial artery.

Measurements of carotid arterial intima-media thickness, an indicator of the presence of atherosclerotic progression, will be assessed using non-invasive B-mode ultrasonography.

Participants will supply a urine sample from the first void (urination) of the morning, for assessment of urinary microalbuminuria using a commercial analysis kit.

*Survey data*

Prior to engaging in the study, all participants will be required to attend their local GP for a regular health check-up to ensure they are appropriate for participation, and complete the following documents*:*

1. Cardiovascular Risk Assessment Form
2. Young Women’s Heart Health Study Survey (to obtain demographic and health information)
3. Food and Activity Record - Information collected from this will be entered into the FoodWorks™ program for a detailed 3-day dietary analysis.
