# Supplementary material for: Effects of a Multi-Disciplinary Lifestyle Intervention on Cardiometabolic Risk Factors in Young Women with Abdominal Obesity: A Randomised Controlled Trial
Source: PLoS One. 2015 Jun 26;10(6):e0130270. doi: 10.1371/journal.pone.0130270 (PMC4483260; doi:10.1371/journal.pone.0130270)

**S3 Text. Lifestyle survey**

**PERSONAL DETAILS**

Your name …………………………………………………………………………………..

Address……………………………………………………………………………………...

Town/Suburb …………………………………………………Postcode…………………...

Phone number/s………………………………. ………………………………………….

Email address………………………………………………………………………………

Date of Birth: …………………………….. Age: ………………………………Years

Height: …………………………………cm Weight: ………………………………kg

**HEALTH STATUS**

In general, would you say your health is: (tick one only)

- Excellent
- Very good
- Good
- Fair
- Poor

In regards to your current weight, how much would you like to weigh now? (circle one only)

5 kg more 1-5 kg more Happy as I am 1-5 kg less 5 kg less

How often have you gone on a diet in order to lose weight during the last year?

- Never
- 1 to 4 times
- 5 to 10 times
- More than 10 times
- I am always on a diet to lose weight

Have you had an illness in the last 12 months that has affected your health, and in particular, your weight?

YES 🞏 NO 🞏

If yes, provide details: ……………………………………………………………………

Have you had any significant accidents in the last 12 months that has affected your health, and in particular you weight?

YES 🞏 NO 🞏

If yes, provide details: ……………………………………………………………………

**MEDICAL HISTORY**

Do you have any medical history of the following conditions? (please tick if applicable)

***Breathing or respiratory conditions***

- Asthma
- Bronchitis
- Other (please specify) ……………………………………………………………

***Blood disorders***

- High blood pressure (hypertension)
- High cholesterol
- Anaemia

***Cardiovascular system***

- Heart disease or vascular conditions
- Stroke
- Chest pain
- Heart rhythm abnormalities
- Other (please specify) ……………………………………………………………

***Reproductive system***

- Polycystic ovarian syndrome
- Other (please specify) ……………………………………………………………

***Metabolic***

- Insulin Dependent Diabetes Mellitus/ Type 1 Diabetes
- Non Insulin Dependent Diabetes Mellitus/ Type 2 Diabetes
- Gestational diabetes (during pregnancy)
- Other (please specify) ……………………………………………………………

***Psychological***

- Depression
- Anxiety disorder
- Insomnia
- Postnatal depression
- Other (please specify) ……………………………………………………………

**MEDICATION USE**

Do you take prescribed medication in the past 6 months?

YES 🞏 NO 🞏

If yes, provide details: ……………………………………………………………………

Has your doctor ever prescribed you medication for your triglyceride levels?

YES 🞏 NO 🞏

If yes, are you taking it now? ………………………………………………………………

Has your doctor ever prescribed you medication for your cholesterol levels?

YES 🞏 NO 🞏

If yes, are you taking it now? ………………………………………………………………

Do you take vitamins, minerals or any other form of health preparations?

YES 🞏 NO 🞏 SOMETIMES 🞏

If yes, provide details: ……………………………………………………………………...

Do you take an oral contraceptive pill, hormone implant or injection?

YES 🞏 NO 🞏

If yes, please specify the brand: ……………………………………………………………

Do you smoke cigarettes/cigars?

YES 🞏 NO 🞏 PREVIOUSLY 🞏

If yes, how many per day: ………………………………………………………………….

If you no longer smoke indicate when you stopped: ………………………………………

**FAMILY HISTORY**

Has any of your close relative (parents, grandparents, aunt / uncles, siblings) suffered from the following (If yes: state their relationship to you):

YES NO Don’t RELATIONSHIP/S

Know (Can be more than one)

1. Obesity 🞏 🞏 🞏 ………………….....
2. Diabetes 🞏 🞏 🞏 ………………….....
3. High blood pressure 🞏 🞏 🞏 ………………….....
4. High cholesterol 🞏 🞏 🞏 ………………….....
5. Heart disease 🞏 🞏 🞏 ………………….....
6. Stroke 🞏 🞏 🞏 ………………….....
7. Mental disorder 🞏 🞏 🞏 ………………….....
8. Any other known hereditary diseases 🞏 🞏 🞏 ………………….....

(Specify: …………………………….)

**PHYSICAL ACTIVITY**

1. Please complete the following table to help us understand the activity patterns that you have done during the past week:

| **Day of the week** | **Type of activity**  (e.g. cycling, swimming, walking) | **Where the activity happened, and who it was with** | **How long the activity went for** | **Approximate intensity**  (e.g. easy, moderate, hard) |
| --- | --- | --- | --- | --- |
| **Monday** |  |  |  |  |
| **Tuesday** |  |  |  |  |
| **Wednesday** |  |  |  |  |
| **Thursday** |  |  |  |  |
| **Friday** |  |  |  |  |
| **Saturday** |  |  |  |  |
| **Sunday** |  |  |  |  |

**Reference:**

Sallis, J., Buono, M., Roby, J., Micale, F., & Nelson, J. (1993). Seven-day recall and other physical activity self—reports in children and adolescents. *Medicine and science in Sport and Exercise, 25*(1), 99-108.

1. How important is it for you to set time aside for regular

physical activity? (tick one box please)

| **Very important** | **Important** | **Neither important nor unimportant** | **Unimportant** | **Very unimportant** |
| --- | --- | --- | --- | --- |
| □ | □ | □ | □ | □ |

1. Please read each of the following statements and indicate which best describes your current exercising habits.

Note: regular exercise = 3 or more times per week for 20 minutes or more each time

|  | **YES** | **NO** |
| --- | --- | --- |
| I currently do NOT exercise, and I do NOT intend to start exercising in the next 6 months | ⬜ | ⬜ |
| I currently do NOT exercise, but I am thinking about starting in the next 6 months | ⬜ | ⬜ |
| I currently exercise some, but not regularly | ⬜ | ⬜ |
| I currently exercise regularly, but I have only begun in the last 6 months | ⬜ | ⬜ |
| I currently exercise regularly, and I have done so for longer than 6 months | ⬜ | ⬜ |
| I have exercised regularly in the past, but I am not doing so regularly | ⬜ | ⬜ |

**Reference:**

Marcus, B., Selby, V., Niara, R., & Rossi, J. (1992). Self-efficacy and the stages of exercise behavior change*. Research Quarterly for Exercise and Sport, 63 (1)*, 60-66.

**NUTRITION INFORMATION**

1. On average, how many times per month, per fortnight, per week or per day do you drink the following beverages?

| **ALCOHOLIC BEVERAGES** | **Never** | **1-2 times per month** | **1-2 times per f/night** | **1-3 time per week** | **4-6 times per week** | **1 time per day** | **2-3 times per day** | **4+ times per day** |
| --- | --- | --- | --- | --- | --- | --- | --- | --- |
| Beer (low alcohol) | ⬜ | ⬜ | ⬜ | ⬜ | ⬜ | ⬜ | ⬜ | ⬜ |
| Beer (full strength) | ⬜ | ⬜ | ⬜ | ⬜ | ⬜ | ⬜ | ⬜ | ⬜ |
| Red wine | ⬜ | ⬜ | ⬜ | ⬜ | ⬜ | ⬜ | ⬜ | ⬜ |
| White wine (incl. sparkling wine) | ⬜ | ⬜ | ⬜ | ⬜ | ⬜ | ⬜ | ⬜ | ⬜ |
| Fortified wines, port, sherry etc. | ⬜ | ⬜ | ⬜ | ⬜ | ⬜ | ⬜ | ⬜ | ⬜ |
| Spirits, liqueurs | ⬜ | ⬜ | ⬜ | ⬜ | ⬜ | ⬜ | ⬜ | ⬜ |

| **HOT BEVERAGES** | **Never** | **1-2 times per month** | **1-2 times per f/night** | **1-3 time per week** | **4-6 times per week** | **1 time per day** | **2-3 times per day** | **4+ times per day** |
| --- | --- | --- | --- | --- | --- | --- | --- | --- |
| Coffee | ⬜ | ⬜ | ⬜ | ⬜ | ⬜ | ⬜ | ⬜ | ⬜ |
| Decaffeinated coffee | ⬜ | ⬜ | ⬜ | ⬜ | ⬜ | ⬜ | ⬜ | ⬜ |
| Tea | ⬜ | ⬜ | ⬜ | ⬜ | ⬜ | ⬜ | ⬜ | ⬜ |
| Herbal tea | ⬜ | ⬜ | ⬜ | ⬜ | ⬜ | ⬜ | ⬜ | ⬜ |
| Chi latte | ⬜ | ⬜ | ⬜ | ⬜ | ⬜ | ⬜ | ⬜ | ⬜ |
| Hot chocolate | ⬜ | ⬜ | ⬜ | ⬜ | ⬜ | ⬜ | ⬜ | ⬜ |

| **COLD BEVERAGES** | **Never** | **1-2 times per month** | **1-2 times per f/night** | **1-3 time per week** | **4-6 times per week** | **1 time per day** | **2-3 times per day** | **4+ times per day** |
| --- | --- | --- | --- | --- | --- | --- | --- | --- |
| Regular carbonated soft drink | ⬜ | ⬜ | ⬜ | ⬜ | ⬜ | ⬜ | ⬜ | ⬜ |
| Diet carbonated soft drink | ⬜ | ⬜ | ⬜ | ⬜ | ⬜ | ⬜ | ⬜ | ⬜ |

1. On average, how many times per month, per fortnight, per week or per day do you eat the following:

| **CEREAL-BASED FOODS** | **Never** | **1-2 times per month** | **1-2 times per f/night** | **1-3 time per week** | **4-6 times per week** | **1 time per day** | **2-3 times per day** | **4+ times per day** |
| --- | --- | --- | --- | --- | --- | --- | --- | --- |
| Porridge | ⬜ | ⬜ | ⬜ | ⬜ | ⬜ | ⬜ | ⬜ | ⬜ |
| Breakfast cereal | ⬜ | ⬜ | ⬜ | ⬜ | ⬜ | ⬜ | ⬜ | ⬜ |
| Rice | ⬜ | ⬜ | ⬜ | ⬜ | ⬜ | ⬜ | ⬜ | ⬜ |
| Pasta or noodles (including lasagna) | ⬜ | ⬜ | ⬜ | ⬜ | ⬜ | ⬜ | ⬜ | ⬜ |
| Crackers, crispbread, dry biscuits | ⬜ | ⬜ | ⬜ | ⬜ | ⬜ | ⬜ | ⬜ | ⬜ |
| Sweet biscuits | ⬜ | ⬜ | ⬜ | ⬜ | ⬜ | ⬜ | ⬜ | ⬜ |
| Cakes, tarts or sweet pastries | ⬜ | ⬜ | ⬜ | ⬜ | ⬜ | ⬜ | ⬜ | ⬜ |

| **DAIRY FOODS and FATS** | **Never** | **1-2 times per month** | **1-2 times per f/night** | **1-3 time per week** | **4-6 times per week** | **1 time per day** | **2-3 times per day** | **4+ times per day** |
| --- | --- | --- | --- | --- | --- | --- | --- | --- |
| Margarine or butter | ⬜ | ⬜ | ⬜ | ⬜ | ⬜ | ⬜ | ⬜ | ⬜ |
| Oil (for cooking) | ⬜ | ⬜ | ⬜ | ⬜ | ⬜ | ⬜ | ⬜ | ⬜ |
| Salad dressing | ⬜ | ⬜ | ⬜ | ⬜ | ⬜ | ⬜ | ⬜ | ⬜ |
| Mayonnaise | ⬜ | ⬜ | ⬜ | ⬜ | ⬜ | ⬜ | ⬜ | ⬜ |
| Ricotta or cottage cheese | ⬜ | ⬜ | ⬜ | ⬜ | ⬜ | ⬜ | ⬜ | ⬜ |
| All other cheeses | ⬜ | ⬜ | ⬜ | ⬜ | ⬜ | ⬜ | ⬜ | ⬜ |
| Cream or sour cream | ⬜ | ⬜ | ⬜ | ⬜ | ⬜ | ⬜ | ⬜ | ⬜ |
| Ice-cream | ⬜ | ⬜ | ⬜ | ⬜ | ⬜ | ⬜ | ⬜ | ⬜ |
| Yoghurt | ⬜ | ⬜ | ⬜ | ⬜ | ⬜ | ⬜ | ⬜ | ⬜ |
| Milkshake or thick-shake | ⬜ | ⬜ | ⬜ | ⬜ | ⬜ | ⬜ | ⬜ | ⬜ |
| Custard | ⬜ | ⬜ | ⬜ | ⬜ | ⬜ | ⬜ | ⬜ | ⬜ |

1. On average, how many times per month, per fortnight, per week or per day do you eat the following:

| **MEATS and FISH** | **Never** | **1-2 times per month** | **1-2 times per f/night** | **1-3 time per week** | **4-6 times per week** | **1 time per day** | **2-3 times per day** | **4+ times per day** |
| --- | --- | --- | --- | --- | --- | --- | --- | --- |
| Beef or veal | ⬜ | ⬜ | ⬜ | ⬜ | ⬜ | ⬜ | ⬜ | ⬜ |
| Chicken | ⬜ | ⬜ | ⬜ | ⬜ | ⬜ | ⬜ | ⬜ | ⬜ |
| Lamb | ⬜ | ⬜ | ⬜ | ⬜ | ⬜ | ⬜ | ⬜ | ⬜ |
| Pork | ⬜ | ⬜ | ⬜ | ⬜ | ⬜ | ⬜ | ⬜ | ⬜ |
| Sausages or frankfurts | ⬜ | ⬜ | ⬜ | ⬜ | ⬜ | ⬜ | ⬜ | ⬜ |
| Processed meats (e.g ham, slami) | ⬜ | ⬜ | ⬜ | ⬜ | ⬜ | ⬜ | ⬜ | ⬜ |
| Bacon | ⬜ | ⬜ | ⬜ | ⬜ | ⬜ | ⬜ | ⬜ | ⬜ |
| Fried fish | ⬜ | ⬜ | ⬜ | ⬜ | ⬜ | ⬜ | ⬜ | ⬜ |
| Steamed, grilled or backed fish | ⬜ | ⬜ | ⬜ | ⬜ | ⬜ | ⬜ | ⬜ | ⬜ |
| Tinned fish (e.g salmon, tuna) | ⬜ | ⬜ | ⬜ | ⬜ | ⬜ | ⬜ | ⬜ | ⬜ |

| **MISCELLANEOUS FOODS** | **Never** | **1-2 times per month** | **1-2 times per f/night** | **1-3 time per week** | **4-6 times per week** | **1 time per day** | **2-3 times per day** | **4+ times per day** |
| --- | --- | --- | --- | --- | --- | --- | --- | --- |
| Pizza | ⬜ | ⬜ | ⬜ | ⬜ | ⬜ | ⬜ | ⬜ | ⬜ |
| Pastries with cheese (e.g quiche) | ⬜ | ⬜ | ⬜ | ⬜ | ⬜ | ⬜ | ⬜ | ⬜ |
| Meat pies, pasties, sausage rolls | ⬜ | ⬜ | ⬜ | ⬜ | ⬜ | ⬜ | ⬜ | ⬜ |
| Hamburgers with a bun | ⬜ | ⬜ | ⬜ | ⬜ | ⬜ | ⬜ | ⬜ | ⬜ |
| Chocolate | ⬜ | ⬜ | ⬜ | ⬜ | ⬜ | ⬜ | ⬜ | ⬜ |
| Confectionary | ⬜ | ⬜ | ⬜ | ⬜ | ⬜ | ⬜ | ⬜ | ⬜ |
| Peanuts or peanut butter | ⬜ | ⬜ | ⬜ | ⬜ | ⬜ | ⬜ | ⬜ | ⬜ |
| Other nuts | ⬜ | ⬜ | ⬜ | ⬜ | ⬜ | ⬜ | ⬜ | ⬜ |
| Corn chips or potato chips | ⬜ | ⬜ | ⬜ | ⬜ | ⬜ | ⬜ | ⬜ | ⬜ |
| Jam, marmalade, syrups or honey | ⬜ | ⬜ | ⬜ | ⬜ | ⬜ | ⬜ | ⬜ | ⬜ |
| Vegemite, Promite or Marmite | ⬜ | ⬜ | ⬜ | ⬜ | ⬜ | ⬜ | ⬜ | ⬜ |
| Tomato sauce or ketchup | ⬜ | ⬜ | ⬜ | ⬜ | ⬜ | ⬜ | ⬜ | ⬜ |

**Reference:**

The cancer Council Victoria. Dietary Questionnaire for Epidemiological studies (Version 3.1).

1. How much do you agree or disagree with the following statements:

**Reference::**

Fowles, E., & Feucht, J. (2004). Testing the Barriers to Healthy Eating Scale. *Western Journal of Nursing Research, 26*(4), 429-443.

**FOOD and BEVERAGE RECALL**

Instructions:

- This food recall is to be completed on two consecutive weekdays and one weekend day (either Saturday or Sunday)
- Please write down everything you eat and drink for the same three days that you keep your activity record
- This is not a test. There are no right or wrong answers. Please do not report the foods you think you should be eating
- Fill in the date and day of the week at the top of the record sheet
- Use as many pages as you need for each day’s record (number each page)
- Start a new page for a new day

## Column 1 – Time

- Every time you have something to eat or drink, write down the time you started
- Write down « am » for morning and « pm » for afternoon or evening

## Column 2 – What you are measuring

Name and full description of all food and drink.

- Write down everything you eat and drink (This include snacks, water, vitamins and mineral supplements).
- For each food and drink use a new line
- Measure each food individually, for example, bread and margarine are each separate foods and are recorded on separate lines.
- Always record cooking methods such as boiling, frying, etc.
- Write down a cut of meat, that is lamb loin chop, chicken leg, rump steak etc.
- Write down if the fat on meat or skin on chicken was eaten or not eaten
- Give a detailed description of the food or drink and brand names, for example : Arnott’s Milk arrowroot Biscuit; Tip Top White Bread

## Column 3 – Amount eaten

- In order to get the best estimate of your nutrient intake we need an accurate estimate of quantities of food and drink consumed.
- Estimate everything as accurately as possible in either **metric cups or spoonfuls** eg teaspoons, tablespoons (level or rounded) such as for breakfast cereal, rice, vegetables or spaghetti, or use a **metric measuring tape or ruler** to give length and width such as for sausage rolls, bananas, etc.

# Recipes:

This includes mashed potato, mixed vegetables dishes, gravies and sauces.

- On a separate page record the individual ingredient with quantities. Report the total amount made and the amount of total recipe consumed. See example attached on blue paper.

# Eating out:

- Estimate food eaten as described above.
- Record the main ingredients in the food if recipe is unknown.
- Record where the food came from, such as McDonald’s.
- Record weights on wrappers, drink cans and other food containers.

# Drinks:

- Measure these in **metric cups or in litre measurements**.
- For cordial, measure the volume of cordial concentrate first then the volume of water added.
- If diluting fruit juice, measure fruit juice and water separately.

**Example**

| **Column 1**  Time | **Column 2**  Name, type, brand cooking method | **Column 3**  Amount | **Leave blank** |
| --- | --- | --- | --- |
| 7.30am | Cornflakes, Kellogs | 1 cup |  |
|  | Lite white milk, Dairy Farmers | ½ cup |  |
|  | White sugar, CSR | 1 teaspoon |  |
| 11.00am | Chocolate Big M milk | 300 ml |  |
| 12.30pm | 1 cheese sandwich |  |  |
|  | White bread, Sunblest | 2 slices |  |
|  | Margarine, unsalted, spread thinly |  |  |
|  | Cheese, Kraft | 1 slice |  |
|  | Uncle Toby’s muesli bar | 31 grams |  |
|  | 1 banana | 12 cm long |  |
|  | Sultanas | ½ cup |  |
| 4.00pm | 1 junior burger, McDonalds |  |  |
|  | 1 small diet coke, McDonalds |  |  |
| 7.00pm | Spaghetti bolognese |  |  |
|  | Boiled spaghetti, no frills | 2 cups |  |
|  | Bolognese sauce (this goes in recipe section) |  |  |
|  | Parmesan cheese, Kraft | 1 teaspoon |  |
|  | Orange juice, Berri | 1 glass |  |
| 8.30pm | Wheatmeal biscuits, Arnotts | 2 |  |
|  | Tea (black), Tetley | 1 cup |  |
|  | White sugar, CSR | 1 teaspoon |  |


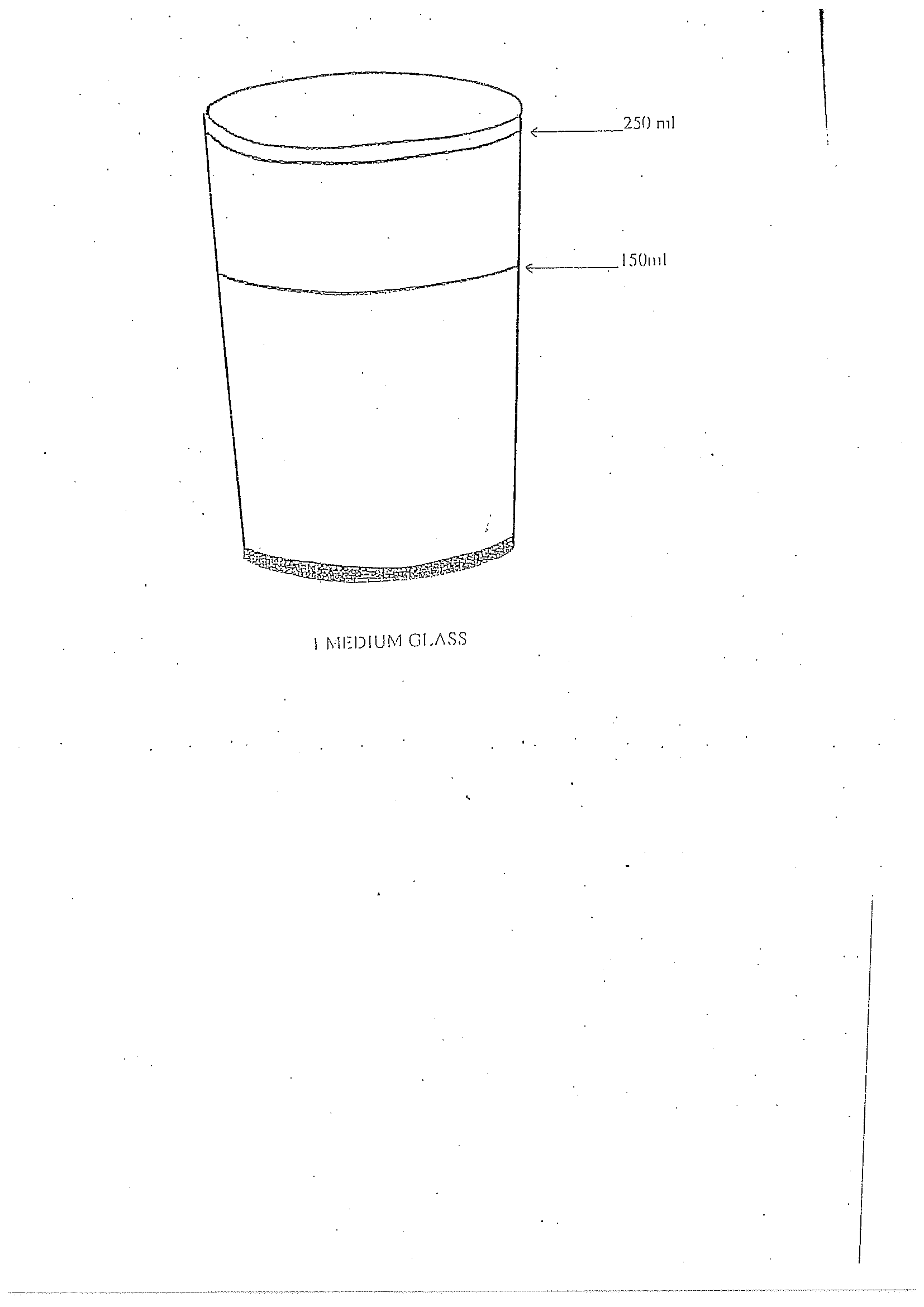
Please use the images on the following pages to best estimate your portion sizes


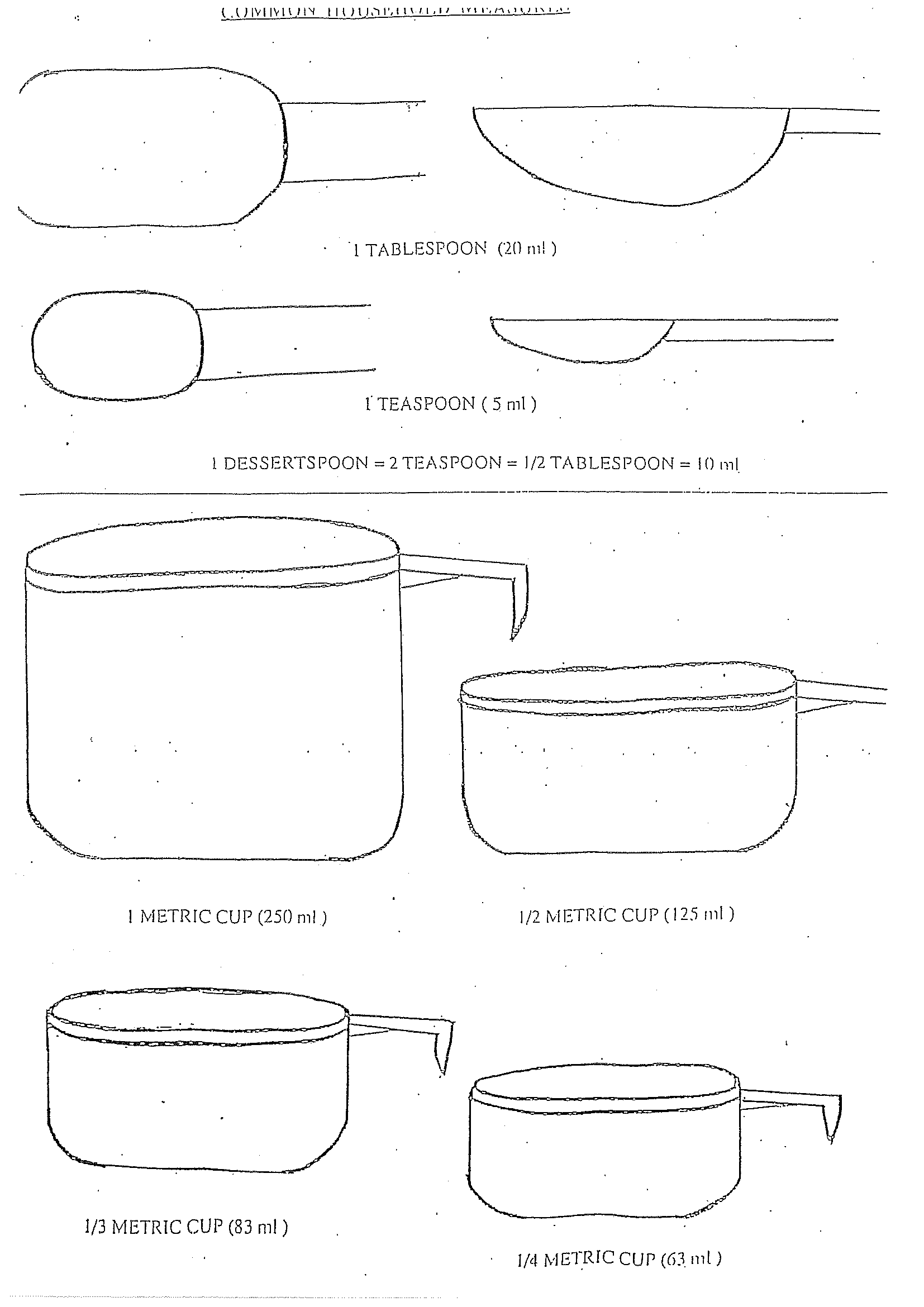


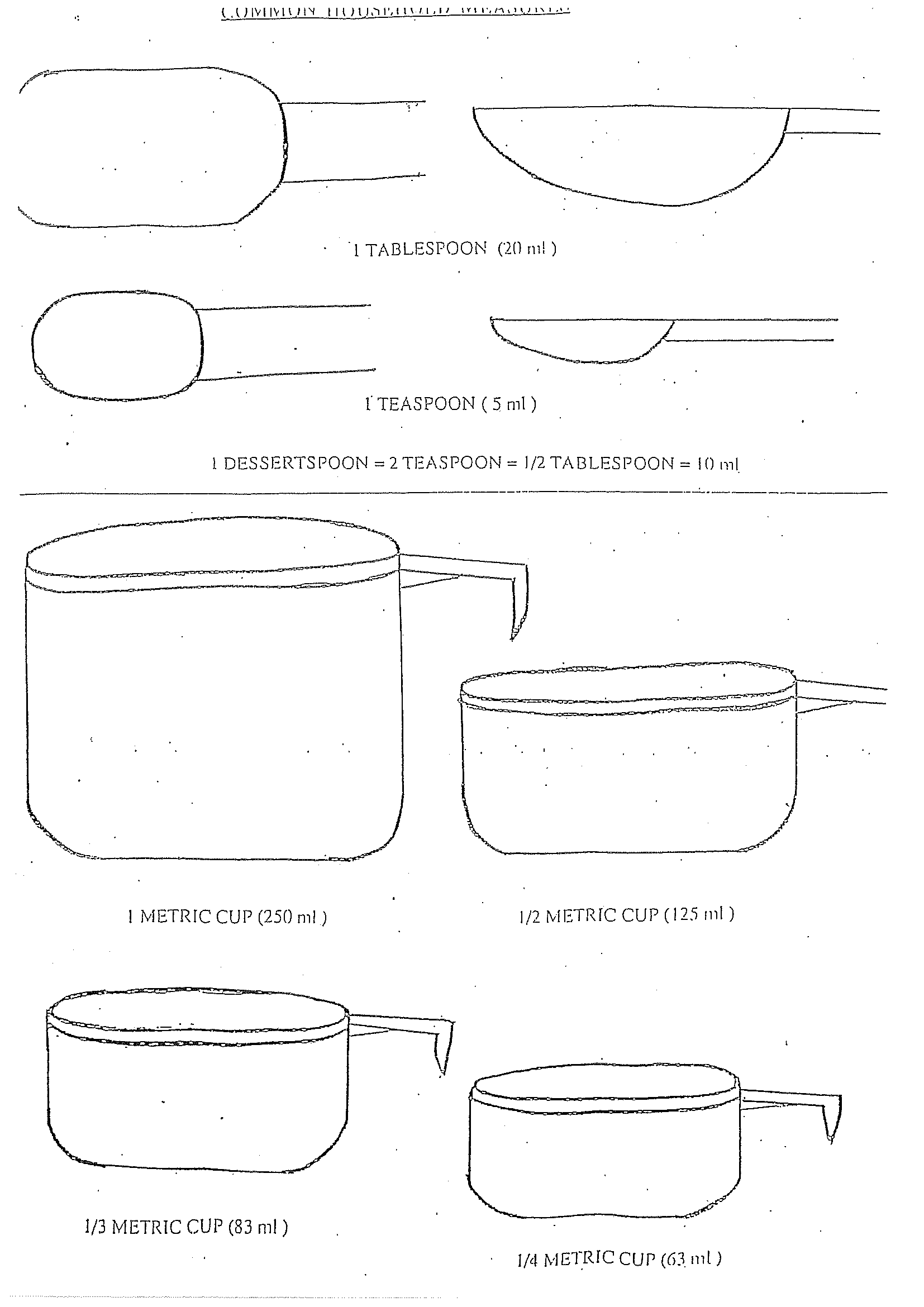

Supplement: S3 Text — (DOC) [file pone.0130270.s005.doc]
